# Local and Global Order in Dense Packings of Semi-Flexible Polymers of Hard Spheres

**DOI:** 10.3390/polym15030551

**Published:** 2023-01-20

**Authors:** Daniel Martínez-Fernández, Miguel Herranz, Katerina Foteinopoulou, Nikos Ch. Karayiannis, Manuel Laso

**Affiliations:** Institute for Optoelectronic Systems and Microtechnology (ISOM) and Escuela Técnica Superior de Ingenieros Industriales (ETSII), Universidad Politécnica de Madrid (UPM), José Gutiérrez Abascal 2, 28006 Madrid, Spain

**Keywords:** semi-flexible polymers, hard sphere, athermal chain, Monte Carlo, molecular simulation, crystallization, packing, phase transition, order parameter, liquid crystal, nematic order, oblate mesogen, prolate mesogen, face centered cubic, hexagonal close packed, bending angle, freely-jointed model, rod-like molecules

## Abstract

The local and global order in dense packings of linear, semi-flexible polymers of tangent hard spheres are studied by employing extensive Monte Carlo simulations at increasing volume fractions. The chain stiffness is controlled by a tunable harmonic potential for the bending angle, whose intensity dictates the rigidity of the polymer backbone as a function of the bending constant and equilibrium angle. The studied angles range between acute and obtuse ones, reaching the limit of rod-like polymers. We analyze how the packing density and chain stiffness affect the chains’ ability to self-organize at the local and global levels. The former corresponds to crystallinity, as quantified by the Characteristic Crystallographic Element (CCE) norm descriptor, while the latter is computed through the scalar orientational order parameter. In all cases, we identify the critical volume fraction for the phase transition and gauge the established crystal morphologies, developing a complete phase diagram as a function of packing density and equilibrium bending angle. A plethora of structures are obtained, ranging between random hexagonal closed packed morphologies of mixed character and almost perfect face centered cubic (FCC) and hexagonal close-packed (HCP) crystals at the level of monomers, and nematic mesophases, with prolate and oblate mesogens at the level of chains. For rod-like chains, a delay is observed between the establishment of the long-range nematic order and crystallization as a function of the packing density, while for right-angle chains, both transitions are synchronized. A comparison is also provided against the analogous packings of monomeric and fully flexible chains of hard spheres.

## 1. Introduction

Over the last few decades, developments in the synthesis of novel polymers and the fabrication of polymer-based materials have turned them into key components of our daily lives. The research is ever-growing in the pursuit of polymer-based materials with enhanced properties as chain connectivity endows macromolecules with unique properties compared to monoatomic systems [1,2]. Describing polymer conformations and configurations statistically and understanding how these are connected to macroscopic properties are thus of paramount importance in technology and industry [3,4].

In parallel, many aspects of the phase behavior and self-organization of general atomic and particulate systems remain unknown or poorly understood. The analysis becomes a great deal harder when macromolecular systems are tackled. Advances in experimental, theoretical, and simulation methods continually enrich our fundamental knowledge of the phenomenon in a wide range of physical systems [5,6,7,8,9,10,11]. For example, theoretical models have been developed to predict the effect of the processing conditions on the phase transition in order to analyze the final morphologies [12,13]. Advances in the synthesis and characterization of colloidal and granular systems have provided significant insights on the phase behavior of monomeric and polymeric systems, particularly given their simplicity and large size compared to traditional polymers [14,15,16,17,18,19,20,21,22]. Simulations can further aid in the research studies of phase transition given the persistent advances at the level of hardware and software, with traditional approaches at the molecular level being based on the Molecular Dynamics (MD) or Monte Carlo (MC) algorithms [23,24,25,26,27].

The hard-sphere model is frequently used in the study of crystallization due to its simplicity and athermal nature, despite the obvious disadvantage of lacking chemical detail. Through molecular dynamics (MD) simulations, early and pioneering works have reported the entropy-driven crystallization of systems of hard spheres [12,28] once the melting point $\varphi_{monomers}^{M}=0.545$ is reached [29] and given sufficient time for the observation of the phenomenon [30,31]. For individual hard spheres, the crystal expected to be obtained in experiments or simulations should be the face centered cubic (FCC), as it is slightly more thermodynamically stable than the hexagonal close-packed (HCP) one [32,33,34]. However, extensive experimental [35,36,37,38,39,40], analytical and simulation [30,41,42,43,44,45] studies have clearly demonstrated the difficulty of obtaining a perfect crystal. Thus, random hexagonal close packing (RHCP) is almost always observed due to the small free energy of stacking faults [46], while the more stable FCC structure appears after a very slow transition from the RHCP morphology [47,48,49,50]. Therefore, crystal perfection in the form of pure FCC crystals is rarely encountered in computer simulations under constant volume and starting from a predominantly amorphous initial configuration [50,51,52].

The mechanism of the crystallization of hard colloidal polymers [52,53,54,55,56,57,58,59,60] differs substantially from that of traditional polymers, the latter as revealed in x-ray scattering studies of short alkane chains [61,62,63], and in MD [64,65,66,67] or MC [54,68] simulations, including simulations with highly precise, all-atom interaction potentials [69,70,71,72,73,74,75,76,77,78]. Just as for monomeric packings, off-lattice MC simulations have shown that random packings of fully flexible linear chains of tangent hard spheres are able to crystallize at high volume fractions through an entropy-driven mechanism, very similar to the one of monomeric analogs [52,55,56,57,79]. Extremely long simulations [52] have further shown the FCC polymorph to be the stable one for fully flexible chains of tangent hard spheres. In shorter simulations, an RHCP phase with a unique stacking direction [55,56,57] appears most often. In spite of the similarities to monomeric counterparts, athermal polymer crystallization shows unique characteristics: the critical volume fraction for the phase transition (melting point) for the fully flexible (freely-jointed, FJ) chains of tangent hard spheres, $\varphi_{chains}^{M}\left(\mathrm{FJ}\right)$ (>0.56), is higher than the melting point of monomers. Monomeric systems present diverse crystal morphologies, ranging between single FCC or HCP structures and close-stacked packings with random directions where twin defects, associated with the formation of fivefold (FIV) symmetry structures, kinetically frustrate crystallization [80,81]. However, both the FCC and RHCP crystals of tangent hard-sphere chains are usually free of twin defects due to a conformational entropic barrier [82]. It has been established that the phase transition and the ordered morphologies of hard-sphere chains are affected insignificantly by chain length, but are sensitive to factors such as gaps in bond lengths [58,83], the presence of surfaces/confinement [59,60,84] and chain stiffness [85,86,87,88].

For semi-flexible polymers [89,90,91,92], the rigidity of the chains is generally implemented in molecular simulations through bending [93,94,95,96,97,98,99,100] and/or torsional [99] potentials. A common topic of study about the phase behavior of semi-flexible polymers is the orientational (nematic) ordering of the chains. According to Onsager, thin and infinitely long rigid rods undergo a transition from the isotropic to the nematic phase, which is driven by entropy, resulting from the competition between translational and orientational ordering [101]. Building on this, Bolhuis and Frenkel mapped out the complete phase diagram of hard spherocylinders as a function of shape anisotropy [102]. The long-range, nematic order in solutions of semi-flexible polymers depends on the size ratio of the chain [103,104,105]. For tangent hard-sphere chains in the rod limit, the transition occurs at high concentrations, still lower than the solidification of the system [106,107]. Theories have been developed [108,109,110,111] and studies have been conducted [112,113,114,115,116,117] under various conditions on the isotropic-to-nematic transition of semi-flexible chains, as the effect on the nematic order and the phase separation of different types of blends through experimental studies [118,119,120] and computer simulations [117,121,122,123,124]. Nguyen et al. studied the effect of chain stiffness and temperature on the competition between crystallization and glass formation for unentangled semi-flexible polymer melts [85,86]. Shakirov and Paul studied the crystallization in melts of short, semi-flexible hard-sphere chains employing Monte Carlo simulations [87,88]. In their works, rod-like polymer melts undergo a first-order transition at a density lower than a melt of hard-sphere monomers or flexible hard-sphere chains [88]. During crystallization, the orientational ordering (nematic) transition is accompanied by a 2-D translational ordering on the plane perpendicular to the chains. These orderings induce the formation of hexagonal crystal planes [89].

Although rod-like chains have been extensively researched in the literature, the corresponding body of work on semi-flexible chains with different bending angles is very limited. Recent MD simulations, studying the jamming and solidification of semi-flexible polymers employing the freely-rotating model varying the fixed bending angle, have revealed the different mechanisms in solidification depending on the equilibrium bending angle, $\theta_{0}$ [125,126].

The present manuscript analyzes the phase behavior of linear, semi-flexible chains of tangent hard spheres, as a function of the equilibrium bending angle and packing density. This is achieved through extensive Monte Carlo (MC) simulations using the Simu-D simulator-descriptor [127], built around polymer-oriented algorithms and particularly chain-connectivity-altering moves (CCAMs) [128,129]. Motivated by previous studies on the crystallization of linear, fully flexible chains of hard spheres [55,56,57,58,59,130,131], we extend the research through the inclusion of chain stiffness via a bending potential that depends on a bending constant, $k_{\theta }$, and an equilibrium bending angle, $\theta_{0}$. The global order, at the level of the chains, is quantified through the orientational (nematic) order parameter, *q*. The local order, at the level of the monomers, is gauged through the Characteristic Crystallographic Element (CCE) norm descriptor [132,133]. Compiling the results of all the simulated systems, we present a phase diagram that reflects the combined effect of packing density and chain stiffness (equilibrium bending angle) on the local and global order of the semi-flexible systems.

This article is organized as follows: in Section 2 we describe the molecular model and the simulated systems, and we briefly explain the descriptors to gauge the long-range order and local structure; Section 3 presents the results on the phase behavior of the simulated semi-flexible systems; finally, we summarize the main conclusions and discuss the current efforts in Section 4.

## 2. Model and Methods

### 2.1. Molecular Model and Systems studied

Polymers are modeled as linear chains of identical hard spherical monomers with a collision diameter *σ*, which is considered the characteristic length. The systems are composed of *N_at_* monomers distributed in *N_ch_* chains of average chain length of *N_av_* (in monomers). All of the systems consist of *N_ch_* = 100 chains of average chain length *N_av_* = 12, resulting in *N_at_* = 1200. The chains present dispersity in their lengths following a uniform distribution in the range N∈[6, 18] due to the presence of chain-connectivity-altering MC moves (see below).

The non-overlapping condition of the hard monomers is adopted by employing the Hard Sphere (HS) potential to describe all of the non-bonded interactions between the monomers. According to the HS potential, the pair-wise energy, $U_{HS}\left(r_{ij}\right)$, is determined by:

(1) $$U_{HS}\left(r_{ij}\right)=\{\begin{array}{c} \;0,\;\;\;\;\;r_{ij}\geq \sigma \\ \;\infty ,\;\;\;\;r_{ij}<\sigma \end{array}$$

where *r_ij_* is the distance between the centers of the monomers *i* and *j*. Periodic boundary conditions are applied in all dimensions, corresponding to a bulk, unconstraint system. For non-overlapping objects’ packing density (volume fraction), φ, is defined as the total volume occupied by the monomers over the volume of the simulation cell. This is given by:

(2) $$\varphi =\frac{V_{mon}}{V_{cell}}=\frac{\pi }{6}\frac{N_{at}}{V_{cell}}\sigma^{3}$$

where *V_mon_* is the total volume occupied by the monomers and *V_cell_* is the volume of the cubic simulation cell.

**Figure 1:**
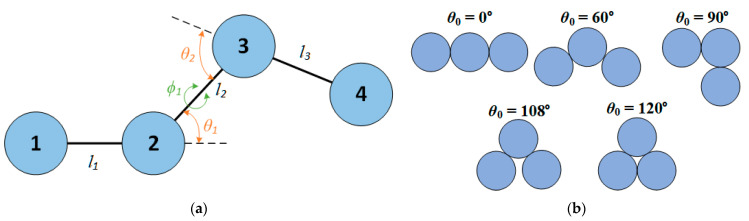
(**a**) Scheme of the bond geometry of a linear sequence of four monomers, indicating bond lengths, *l*, bending angles, *θ*, and torsion angles, ϕ. (**b**) Schematic representation of trimers having equilibrium bending angles ($\theta_{0}$ ) studied in the present work.

Figure 1 shows a sketch of the bond geometry encountered in linear chains, consisting of bond lengths, *l*, bending angles, *θ*, and torsion angles, ϕ. Bond lengths can fluctuate uniformly in the interval l∈[σ, σ+dl], where *dl* is the maximum gap between successive monomers of the chain. For all of the systems simulated here, $dl=6.5\times 10^{-4}$ (in units of *σ*), practically enforcing tangency between bonded monomers [58]. Chain stiffness is introduced through a potential controlling the bending angle, *θ*, which is the angle formed by two successive bond vectors (Figure 1). The bending potential is given by:

(3) $$U_{bend}\left(\theta \right)=k_{\theta }{\left(\theta -\theta_{0}\right)}^{2}$$

where $k_{\theta }$ is the bending constant and $\theta_{0}$ is the equilibrium bending angle. For fixed bond lengths, setting $k_{\theta }=0$ allows the simulation of freely-jointed chains while $k_{\theta }\to \infty$ corresponds to freely-rotating chains. The equilibrium bending angle can vary from fully extended ($\theta_{0}=0{}^{\circ}$) to fully compact ($\theta_{0}=120{}^{\circ}$) configurations of triplets. In this work, we study five specific equilibrium bending angles: 0°, 60°, 90°, 108°, and 120°, as they are represented, schematically, in Figure 1. Torsion angles, ϕ, are allowed to fluctuate freely.

**Figure 2:**
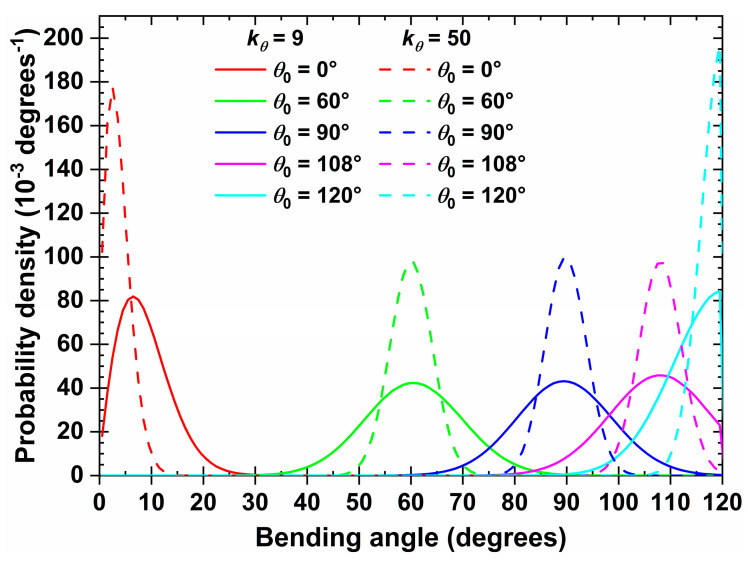
Distribution of bending angle, *θ*, of a 100-chain system of *N_av_* = 12 at φ=0.30 in the bulk for $k_{\theta }=9$ (solid lines) and 50 (dashed lines) and different equilibrium bending angles, $\theta_{0}$.

The bending constant is set equal to $k_{\theta }/k_{B}T=9\;$rad^−2^ (for simplicity in the continuation we will report it as $k_{\theta }=9$) in all of the simulated systems, where $k_{B}$ and *T* are the Boltzmann constant and temperature, respectively. The specific choice of the harmonic constant was made considering the computational expediency and the fact that this range of constraints leads to orientational ordering for rod-like chains, as demonstrated in [87]. This degree of stiffness limits the deviations from the equilibrium bending angle to, approximately, ±20°, as shown in Figure 2 where, for comparison, we also present the corresponding curves as obtained by increasing the bending constant to $k_{\theta }=50$.

### 2.2. Simulation Algorithm

The simulations are carried out by means of the Simu-D simulator-descriptor suite [127]. The simulations are performed under constant volume and temperature ($T=1/k_{B}$), employing Periodic Boundary Conditions (PBCs). The equilibrium simulations are performed mainly at high packing densities in the range φ=[0.54, 0.60]. Additionally, simulations are also carried out at significantly lower volume fractions to study the long-range orientational order, particularly for rod-like chains ($\theta_{0}=0{}^{\circ}$). The choice of volume fractions is driven by the fact that, in past studies, we showed that crystallization for systems of freely-jointed chains of tangent hard spheres occurs once a packing density of φ≈ 0.58 is reached while at φ= 0.56 polymer packings remain amorphous [55,56,57]. For comparison, monomeric analogs crystallize at $\varphi_{monomers}^{M}=0.545$. In Figure S1 (see Supplementary Materials), computer-generated configurations are visualized corresponding to dilute conditions (φ= 0.10) for all of the semi-flexible systems studied here.

The systems are generated and equilibrated by employing a mix of localized algorithms and chain-connectivity-altering moves (CCAMs), as implemented in the Simu-D simulator [127]. The MC mix is composed of the following moves: reptation (10%); rotation (10%); flip (34,8%); intermolecular reptation (25%); end-segment re-arrangement (or CCB as in Refs. [134,135]) (20%); simplified end-bridging (sEB) (0.1%); and simplified intramolecular end-bridging (sIEB) (0.1%). The attempt probabilities of each MC move are reported in parentheses and remain unaltered for all of the systems. Due to the high packing densities, all of the local moves are executed according to a configurational bias (CB) pattern (see more technical explanations in Refs. [127,128]), with the number of trials per attempted move depending on the packing fraction, according to Table 1 of Ref. [128].

The initial configurations are generated at very dilute conditions (φ=0.001) as fully flexible systems. Under these conditions, the desired equilibrium bending angle, $\theta_{0}$, and bending constant, $k_{\theta }$ are activated, followed by rapid constant-volume equilibration. The dilute systems of the semi-flexible chains are then shrunk until the desired volume fraction is reached through isotropic volume compressions attempted at regular intervals [128]. The equilibrium, constant-volume simulations are conducted between a minimum of $5\times 10^{11}$ and a maximum of $8\times 10^{11}$ MC steps, depending on the system. The systems’ configurations (“frames” or “snapshots”) are recorded every $10^{7}$ MC steps.

### 2.3. Global (Long-Range) Orientational Order

As we simulate semi-flexible chains, it is important to quantify the long-range orientational order of the chains [101]. In the nematic order, the chains exhibit a long-range orientation with their long axis aligned along a preferent direction, denominated nematic director ***n***. The isotropic-to-nematic transition is influenced by parameters such as packing density [106,107] and ratio of chain size [103,104,105].

The chain orientational order is determined through averages of a second-order invariant [136]. The orientation of each molecule is defined by the unit vector ***u*** along the long axis of the molecule. The long axis of each chain is determined by the inertia tensor ***I***, a second-order tensor calculated according to Equation (4), where *N(j)* is the number of monomers of the chain *j*, *m_i_* is the mass of the monomer (considered here as unity), ***x****_i_* is the coordinate vector of the monomer, ***x_cm_*** is the coordinate vector of the centre of mass of the chain and ***δ*** is the second order isotropic tensor. The long axis of each chain corresponds to the eigenvector, $v_{3}$, of the lowest eigenvalue of the inertia tensor ***I*** so that the unit vector ***u*** is calculated normalizing the eigenvector $v_{3}$.

(4) $$I=\overset{N\left(j\right)}{\underset{i=1}{\sum }}m_{i}\left({\left\|\frac{x_{i}-x_{cm}}{\sigma }\right\|}^{2}\delta -x_{i}x_{i}\right)$$

(5) $$u=\frac{v_{3}}{\left\|v_{3}\right\|}$$

As an average of all the molecular orientations, we define the second-order tensor ***Q*** according to:

(6) Q=1Nch∑i=1Nchuiui−13δ.

The ***Q*** tensor is: (1) symmetric, and (2) traceless (*Tr**Q*** = 0). These two properties reduce the independent components of the second-order tensor from 9 to 5.

The tensor ***Q*** can be calculated for the different ideal phases (isotropic phase; prolate mesogen, nematic mesophase; and oblate mesogen, nematic mesophase; for their schemes see for example Figure 4 in [136]). For the isotropic phase, denoted here as “ISO”, the molecules can present any random orientation with equal probability, so no orientation is preferred. Thus, all components of the resulting matrix ***Q^ISO^*** are zero. In the perfectly aligned prolate mesogen, the long axis of every molecule is aligned along a preferred orientation leading to the nematic mesophase. In a coordinate system in which the preferred orientation of the system is along the x-axis, the matrix representation of ***Q*** for the perfect prolate mesogen, nematic mesophase, denoted here as “PRO”, takes the form:

(7) QPRO=(23000−13000−13)

In the oblate mesogen, nematic mesophase, it is the short axis of the molecules that tends to be aligned with the nematic director ***n***. In the same coordinate system, the matrix representation of ***Q*** for the oblate mesogen, nematic mesophase, denoted here as “OBL”, takes the form:

(8) QOBL=(−130001600016)

In order to identify the similarities with these ideal cases, we diagonalize ***Q*** and use its normalized eigenvalues as unit vectors of a new Cartesian coordinate system where ***Q’*** is diagonal and its diagonal elements are its eigenvalues (*λ_1_*, *λ_2_*, and *λ_3_*), ordered by decreasing the absolute value. The eigenvector of the largest absolute eigenvalue *λ_1_*, in the case of a nematic mesophase (denoted here as “NEM”), would correspond to the preferred orientation of the system or nematic director ***n***.

A scalar order parameter *q* is obtained by comparing the diagonal tensor ***Q’*** with the corresponding tensor ***Q^PRO^*** of a perfect prolate mesogen, nematic mesophase. This scalar *q* represents the degree of alignment between the chains. In an isotropic system, where ***Q*** is null, *q* → 0. For the perfectly aligned prolate mesogen, nematic mesophase system, q=1, while q=−1/2 for the ideal oblate mesogen, nematic mesophase. The orientational order parameter, thus, takes values in the range −1/2≤q ≤1.

(9) Q′=(λ1000λ2000λ3)=q(23000−13000−13)       |λ1|>|λ2|≥|λ3|

Accordingly, the long-range orientational order is characterized by the scalar orientational order parameter *q* and the nematic director ***n***.

### 2.4. Local Order: Characteristic Crystallographic Element Norm

The metric used in this work to identify the disorder-order transition at the local level and quantify the degree of crystallinity of the simulated systems is the Characteristic Crystallographic Element (CCE) norm, which is explained in detail in Refs. [132,133]. The CCE norm is integrated into the descriptor part of the Simu-D suite, which is also used for the MC simulations [127].

The CCE norm descriptor gauges the crystallinity of an atomic or particulate system in two (2D) or three (3D) dimensions by comparing the local environment around each site with a set of ideal reference crystals under the main concept that each ideal crystal is uniquely identified by a set of symmetry operations [137,138,139,140]. Therefore, for every site *i* of a system, the CCE norm descriptor identifies the nearest neighbors and quantifies the orientational and radial deviations of the “real” local environment with respect to the “ideal” environment of each reference crystal. This comparison provides, for the given site *i,* a CCE norm value with respect to each *X* reference crystal, $\varepsilon_{i}^{X}$. The closer the *X*-CCE norm is to zero, the higher the similarity of the local environment to the respective reference crystal *X*. Site *i* is identified as an *X*-type crystal when the calculated CCE norm is lower than a critical threshold, $\varepsilon_{i}^{X}\leq \varepsilon^{thres}$. In past studies, an empirical threshold of 0.245 was determined for packings of non-overlapping spheres [56,132,133] in the bulk [55,56,57,58,80,81] and under confinement [59,60]. Due to the strict concept behind the descriptor, the CCE norm is highly discriminatory, so the value of a site cannot be simultaneously very low for two different reference crystals. The current version of the CCE norm descriptor can identify similarity with respect to the following crystals: hexagonal close packed (HCP); face centered cubic (FCC); body centered cubic (BCC); and hexagonal (HEX) for 3D systems, and triangular (TRI); square (SQU); and honeycomb (HON) for 2D systems. The fivefold (FIV) and pentagonal (PEN) local symmetries can also be identified in three and two dimensions, respectively. Sites that cannot be assigned to any of the previous reference ideal environments are labeled as amorphous (AMO) or, more precisely, as “unidentified”.

The process explained before is repeated over all sites of the system for each reference crystal. Once the CCE norm has been evaluated for every site and reference crystal, an order parameter for each reference crystal *X*, $S^{X}$ ($S^{X}\in \left[0,1\right]$), can be calculated for the snapshot as:

(10) $$S^{X}=\overset{\varepsilon^{thres}}{\underset{0}{\int }}P\left(\varepsilon^{X}\right)d\varepsilon^{X}$$

where $P\left(\varepsilon^{X}\right)$ is the probability function of CCE-based norms of the reference crystal *X*.

Additionally, a degree of crystallinity (or total crystallinity), $\tau_{c}$, can be calculated as the sum of the order parameters of all the reference crystals. For a bulk, 3D system, the degree of crystallinity is calculated as:

(11) $$\tau_{c}=\overset{4}{\underset{k=1}{\sum }}\;\overset{\varepsilon^{thres}}{\underset{0}{\int }}P\left(\varepsilon^{X}\right)d\varepsilon^{X}=\overset{4}{\underset{k=1}{\sum }}S^{k}=S^{HCP}+S^{FCC}+S^{BCC}+S^{HEX}.$$

In the present work, no appreciable population of sites with HEX or BCC similarity was detected (*S^HEX^*, *S^BCC^* → 0). Thus, in the continuation, we consider only the HCP and FCC crystals, as well as fivefold local symmetry (FIV).

## 3. Results

The phase behavior of polymer chains under the effect of the chain stiffness is the main focus of this work, emphasizing the long-range, orientational (nematic) ordering and the local structure.

**Figure 3:**
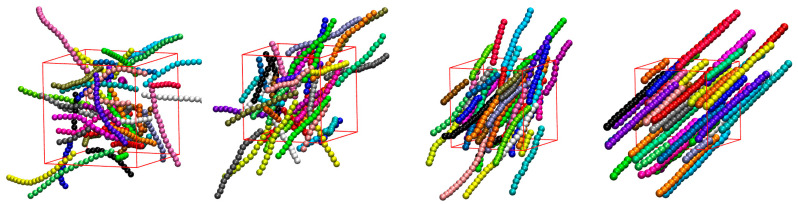
Snapshots of the initial configurations for the 100-chains, *N_av_* = 12, $k_{\theta }=9$, $\theta_{0}=$ 0° system showing at increasing packing densities. From left to right: φ  = 0.10, 0.20, 0.30, and 0.50. Monomers are colored according to the parent chain. For clarity, only 50 chains (half of the total population) are shown. Chains are shown with the coordinates being fully unwrapped in space. Images created with VMD visualization software [141]. Individual panels are also available as stand-alone, interactive, 3-D images (in Supplementary Materials).

Prior to the simulation data being analyzed in a post-processing step, a preliminary visual inspection of the initial and resulting system configurations is performed. In the case of rod-like chains ($\theta_{0}=0{}^{\circ}$), the visual inspection of the initial configurations, as presented in Figure 3, suggests a transition from an isotropic (φ = 0.10 and 0.20) to a nematic phase (φ = 0.30 and 0.50) with increasing packing density, i.e., the rod-like chains align in the system along the common nematic director. This transition occurs at a packing density that is significantly lower than for solidification, in perfect qualitative agreement with past independent works on similar systems [101,106,107]. Figure S2 (see Supplementary Materials) shows snapshots at the end of the simulation for all systems at φ= 0.60.

### 3.1. Global Orientational Order

**Figure 4:**
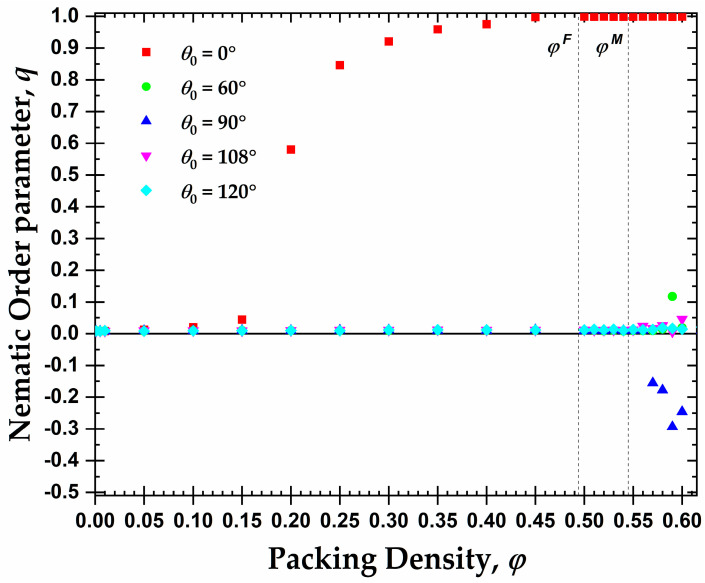
Evolution of the orientational (nematic) order parameter, *q*, as a function of the packing density, φ, at different equilibrium bending angles, $\theta_{0}$. The freezing, $\varphi_{monomers}^{F}\approx 0.495$, and melting, $\varphi_{monomers}^{M}\approx 0.545$, points of monomeric hard spheres are also shown as dashed vertical lines for comparison.

Following the preliminary visual inspection of the initial configurations for the rod-like chains ($\theta_{0}=0{}^{\circ}$), the long-range, orientational (nematic) ordering is analyzed through the second-order tensor ***Q*** and the scalar order parameter *q*, as explained previously. As an example, Figure S3 (see Supplementary Materials) shows the evolution of the exponential moving average of *q* as a function of the MC steps for rod-like chains at different packing densities. The dependence of the nematic order parameter *q* on the packing density, φ, is presented in Figure 4 for all of the semi-flexible systems under study ($\theta_{0}=$ 0, 60, 90, 108, and 120°), averaged over all frames of the equilibrated part of the simulation trajectory. In line with Figure 3, the rod-like chains of average length *N_av_* = 12 show a well-defined isotropic-nematic transition as the concentration increases. This transition takes place in the interval 0.15 ≤ φ ≤ 0.20 and very closely resembles the prediction of Onsager’s theory. At higher volume fractions, the chains form a prolate mesogen, nematic mesophase (PRO), as seen by the monotonically increasing value of *q*. A perfect PRO phase (*q* → 1) is established at packing densities approximately equal to φ = 0.45. For the average length studied here (*N_av_* = 12), the transition from the isotropic to the nematic phase, ISO → PRO (i.e., nematic mesophase with prolate mesogens) for rod-like athermal chains occurs at significantly lower packing densities ($\varphi^{nem}\approx 0.20$) than the freezing ($\varphi_{monomers}^{F}\approx 0.495$) and melting ($\varphi_{monomers}^{M}\approx 0.545$) points of monomeric hard spheres.

In addition to this expected isotropic-to-nematic (ISO → PRO) transition exhibited by the rod-like chains, a nematic mesophase with oblate mesogens, OBL, (*q* < 0) appears for semi-flexible chains with $\theta_{0}=$ 90°. In contrast to the long-range orientational order of the rod-like chains, the isotropic-to-oblate, ISO → OBL, transition takes place at $\varphi^{obl}\approx 0.57$, which is higher than the melting point of HS.

For the remaining values of the equilibrium bending angles, each chain system remains in the isotropic phase in the whole concentration range. Still, a small peak is produced at very high packing densities that can be associated with chains primarily of $\theta_{0}=$ 60°, taking some preferred directions, corresponding to the values of the nematic order parameter of around *q* ≈ 0.15. This can be explained as the establishment of the FCC and HCP crystallites (see later discussion on local order) enforces specific bending angles which are compatible with these crystals. If such bending angles are not available through intra-chain arrangements, then the only other option is inter-chains ones, thus inducing partial alignment among the chains at a local level.

**Figure 5:**
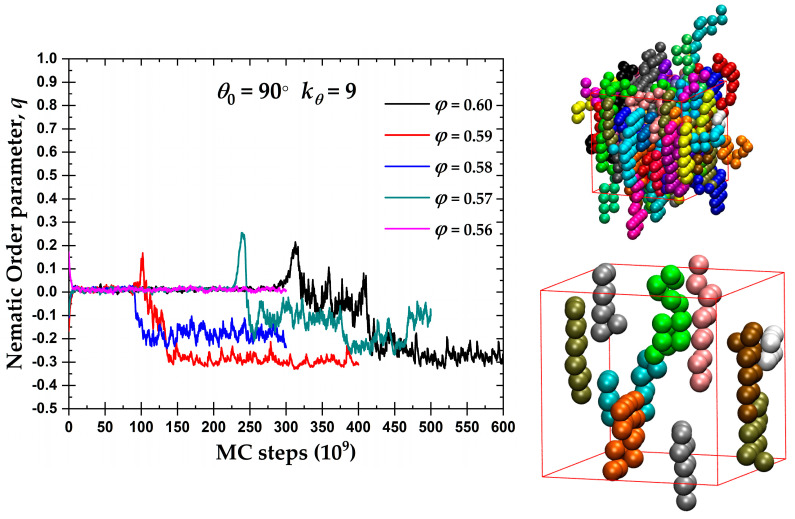
(**Left**) Exponential moving average of the orientational order parameter, *q*, as a function of MC steps for the 100-chains *N_av_* = 12 semi-flexible system with bending constant $k_{\theta }=9$ and equilibrium bending angle $\theta_{0}=90{}^{\circ}$ at different packing densities, φ. (**Right**) Visual representations at φ=0.59 showing all 100 chains (**top**) and 10 randomly selected chains (**bottom**) with coordinates of the monomers being fully unwrapped in space. Monomers are colored according to the parent chain. Images created with VMD visualization software [141]. Individual panels are also available as stand-alone, interactive, 3-D images (in Supplementary Materials).

In the case of semi-flexible chains with $\theta_{0}=90{}^{\circ}$, the left panel of Figure 5 hosts the evolution of the exponential running average of the orientational order parameter, *q*, as a function of the MC steps for the packing densities where a certain degree of nematic order was observed in Figure 4. A tendency for the formation of an oblate mesogen, nematic mesophase (OBL) is observed after enough simulation time. The degree of ordering of the oblate mesogens increases with the volume fraction, as indicated by *q* approaching the ideal value −1/2. Compared to the crystallization at the level of monomers (see below), the ISO → OBL transition for $\theta_{0}=$ 90° takes places with an equal or even slower rate. We should note here that the “rate” used in the present manuscript has no physical meaning and it corresponds to the number of MC steps required to observe the phase transition. Thus, the nematic phase for the semi-flexible chains with $\theta_{0}=$ 90° practically coincides with crystallization, i.e., self-organization at the local and global levels appears synchronized.

The right panels of Figure 5 show the semi-flexible chains with $\theta_{0}=$ 90° at φ=0.59 at the end of the MC simulation, once the system has reached the OBL state. The top snapshot presents the unwrapped representation of the semi-flexible chains of the complete system. As in the previous snapshots, the monomers are color-coded according to the parent chain. For clarity, the bottom snapshot contains only 10 randomly selected chains of the same system. The semi-flexible chains tend to form flat layers, interrupted occasionally by right-angle jumps between planes, consistent with the employed constraint of $\theta_{0}=$ 90°. These flat chain configurations have, in general, a common behavior that further explains the OBL phase in Figure 4 and the left panel of Figure 5.

### 3.2. Local Order: Crystal Nucleation and Growth

The local structure is gauged through the CCE norm descriptor [132,133]. For consistency with our past studies on the flexible chains of hard spheres, we employ a threshold of $\varepsilon^{thres}=0.245$ to label a site as *X*-type, where *X* is the reference crystal or local symmetry. In the present work, given that no appreciable population of BCC or HEX sites is detected in any of the simulated systems, *X* corresponds to HCP, FCC, and FIV. In the continuation, and throughout the manuscript, the corresponding colors to be used for the representation of the HCP, FCC, and FIV sites (in snapshots) and curves (in figures) are blue, red, and green, respectively. The results from the semi-flexible systems are also compared with the ones of fully flexible (freely-jointed) chains of tangent hard spheres simulated and analyzed through the Simu-D software under the same conditions of volume fraction and average chain length [55,56,57,130,131].

**Figure 6:**
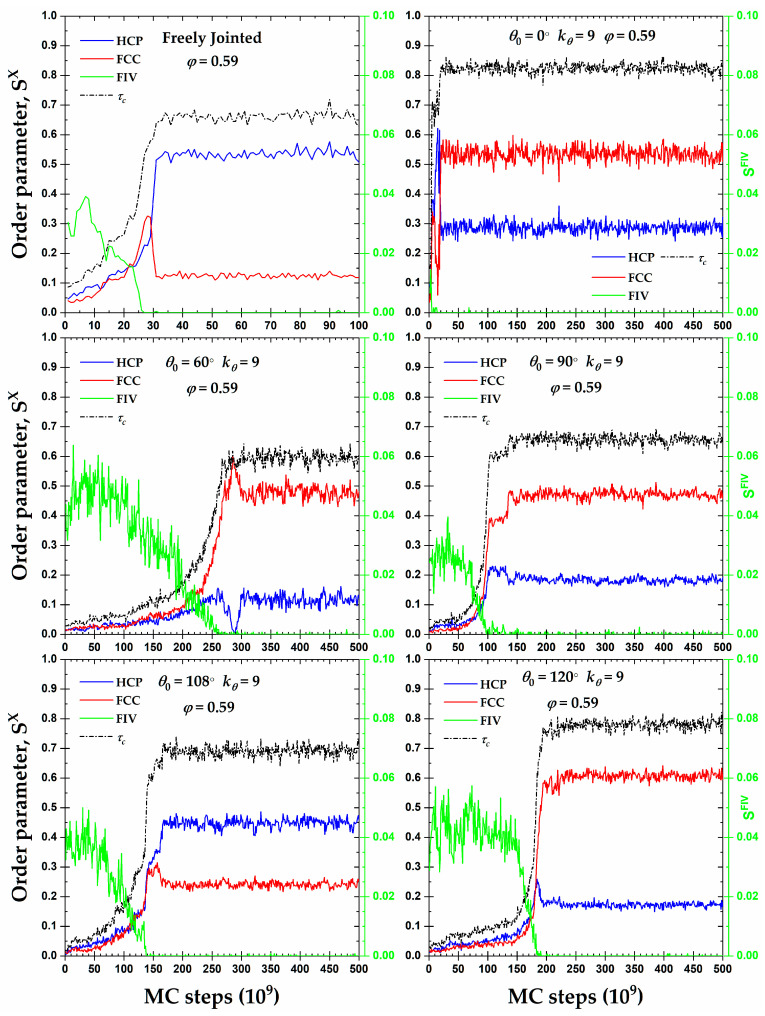
Crystal nucleation and growth, as quantified by the evolution of the HCP, $S^{HCP},$ and FCC, $S^{FCC},\;\mathrm{order}\;\mathrm{parameters}$ ((**left**) *y*-axis), and fraction of fivefold-like sites, $S^{FIV}$ ((**right**) *y-*axis), as a function of MC steps for 100-chains *N_av_* = 12 systems of freely-jointed chains and semi-flexible chains with bending constant $k_{\theta }=9$ and equilibrium bending angle $\theta_{0}=$ 0, 60, 90, 108, and 120° at a packing density φ=0.59.

Figure 6 presents the CCE-based order parameters for HCP ($S^{HCP}$), FCC ($S^{FCC}$), and FIV ($S^{FIV}$), along with the degree of crystallinity, $\tau_{c}$, as a function of the MC steps at a packing density φ=0.59 for all of the semi-flexible systems, including the fully flexible one as reference. This volume fraction is higher than the melting transition of the freely-jointed hard-sphere chains, as established in [52,55,56,57,79]. An inspection of all of the panels shows clearly that the mechanism of the phase transition, here in the form of crystallization, is very similar and rather independent of the equilibrium bending angle. The initial packings are amorphous (AMO) as the fraction of sites with non-ordered local structure is vastly dominant: the percentage of close packed sites does not exceed 5% and, in most cases, the FIV population is commensurate or even exceeds the combined HCP + FCC one. This initial state of the AMO athermal chain packings agrees with the past works on dense monomeric and polymer assemblies [57,60,80,81,82]. Out of all of the systems, the rod-like one is characterized by the lowest population of FIV sites at the beginning, as FIV appears to be incompatible with the perfect nematic ordering exhibited by the chains at this range of volume fractions.

Once crystallites of the HCP and FCC characters start growing, at almost identical rates, the fraction of the FIV-like sites decreases gradually until it practically disappears. The structural competition, observed here for all of the semi-flexible chain systems, between the FIV local symmetry and crystallization in the form of the HCP and FCC sites is in perfect match with identical observations in simulations of fully flexible [52,55,56,57,58,59,60] and monomeric HS systems [80,81], including bead-spring chains under quenching [85,142]. Once the population of FIV sites is eliminated, a trend manifestly valid for all of the simulated packings, the relative fractions of the HCP and FCC crystals undergo a sharp variation, which is followed by the establishment of the final, stable ordered structures of the crystalline (CRY) character. The average degree of crystallinity in the final CRY phase ranges between 0.65 and 0.85, with the lowest and highest values corresponding to the fully flexible and rod-like chains, respectively. At φ=0.59, as seen in Figure 6, all of the crystals contain appreciable fractions of FCC and HCP sites, and no perfection is registered. Accordingly, one expects that the formed morphologies correspond to fivefold-free but defect-ridden RHCP crystals.

**Figure 7:**
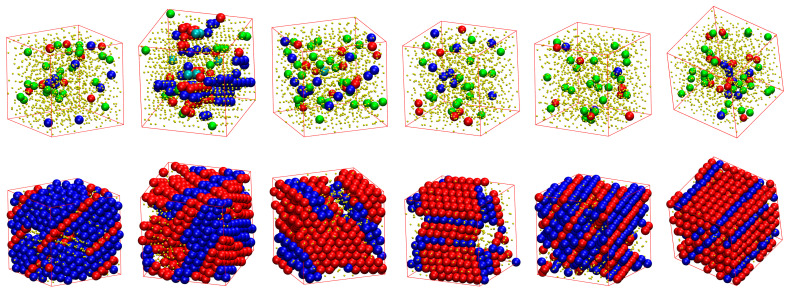
Snapshots of the initial (**top**) and final (**bottom**) configuration of the MC simulations for the 100-chains *N_av_* = 12 systems at φ=0.59 of (from left to right) freely-jointed chains and semiflexible chains ($k_{\theta }=9$ ) with equilibrium bending angle $\theta_{0}=$ 0, 60, 90, 108, and 120°. Monomers are color-coded according to CCE norm. Blue, red, cyan, purple, and green correspond to HCP-, FCC-, BCC, HEX-, and FIV-like sites, respectively. Amorphous (AMO) sites are colored in yellow and are shown with reduced dimensions for visual clarity. Individual panels are also available as stand-alone, interactive, 3-D images (in Supplementary Materials).

Figure 7 shows, for all of the systems whose phase transition is presented in Figure 6, the snapshots at the beginning (top panels) and the end (bottom panels) of the corresponding MC simulations. As already quantified by the data in Figure 6, all of the initial configurations show amorphous structures with a remarkable population of FIV-like sites. At the end of the MC simulations, the crystalline phase of every system shows a stable configuration of mixed HCP/FCC structures, being defect-ridden and fivefold-free, exactly as expected by the fractions of sites in Figure 6. In their majority, the semi-flexible chain packings form RHCP structures of alternating layers of unique FCC and HCP character with a single stacking direction, as in the freely-jointed systems. Additionally, the semi-flexible systems can also form crystal structures with multiple, random stacking directions, as it is observed for the system with $\theta_{0}=90{}^{\circ}$.

The formation of RHCP crystals of mixed HCP/FCC structures, with unique or multiple stacking directions, is also observed for all of the equilibrium bending angles and packing densities studied where crystallization takes place.

**Figure 8:**
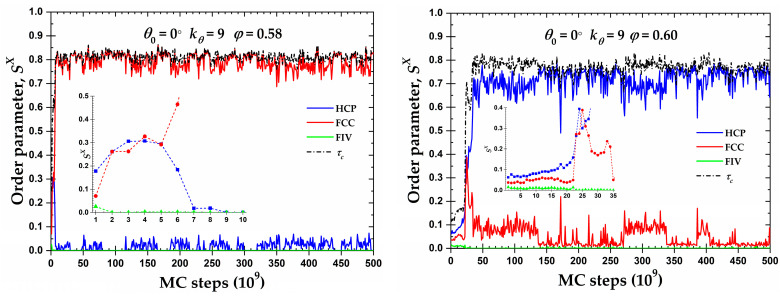
CCE norm order parameter, $S^{X}$, as a function of MC steps for the 100-chains *N_av_* = 12 semi-flexible system chains with bending constant $k_{\theta }=9$ and equilibrium bending angle $\theta_{0}=0{}^{\circ}$ at φ= 0.58 ((**left panel**)) and 0.60 ((**right panel**)). Inset corresponds to the early part of the MC trajectory ((*left*): 10 × 10^9^ and (*right*): 35 × 10^9^ MC steps). Dashed black line corresponds to the degree of crystallinity, $\tau_{c}$.

**Figure 9:**
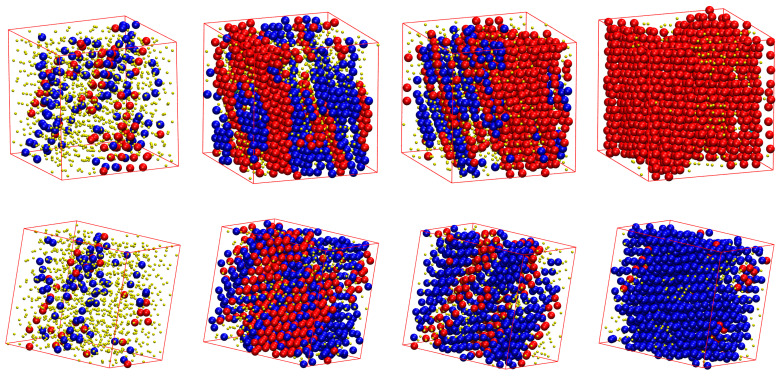
Computer-generated representations of the crystal growth of the 100-chains *N_av_* = 12 semi-flexible system chains with bending constant $k_{\theta }=9$ and equilibrium bending angle $\theta_{0}=0{}^{\circ}$ at (**top**) φ=0.58 after (from left to right) 1, 25, 60 and $100\times 10^{8}$ MC steps, and at (**bottom**) φ=0.60 after (from left to right) 1, 240, 300 and $350\times 10^{8}$ MC steps. Monomers are color-coded according to CCE norm. Blue, red, and green correspond to HCP-, FCC-, and FIV-like sites, respectively. Amorphous (AMO) sites are colored yellow. For visual clarity, monomers are shown with reduced dimensions. Individual panels are also available as stand-alone, interactive, 3-D images (in Supplementary Materials).

Although almost all of the semi-flexible systems crystallize in RHCP crystals of mixed HCP/FCC layers, two important exceptions exist for the rod-like chains ($\theta_{0}=0{}^{\circ}$) at φ= 0.58 and 0.60. Figure 8 shows the CCE-based local order parameter, $S^{X}$, and the total crystallinity, $\tau_{c}$, as a function of the MC steps, while the corresponding snapshots at various simulation instances can be found in Figure 9. Both systems show very similar trends: after a very short initial period, characterized by the rapid increase in the HCP and FCC sites and the parallel reduction of the FIV population, the growth rates stop being the same and one type grows in favor of the other. At φ=0.58, the resulting morphology is an almost perfect FCC crystal ($\tau_{c}=0.83$, $S^{FCC}=0.83$), while the opposite occurs at φ=0.60, where a (less) perfect HCP crystal emanates ($\tau_{c}=0.77$, $S^{HCP}=0.74$). In the case of the HCP crystal at φ=0.60, the resulting structure is not as stable as that of the FCC crystal, alternating between the almost perfect HCP crystal ($S^{FCC}\to 0$) and an HCP crystal with a small population of FCC-like sites ($S^{FCC}\approx 0.1$). For the latter case, when FCC impurities appear in the HCP crystal, these are produced on the border of the crystal with a regime of defects in the form of amorphous (AMO) sites. Figure S4 (see Supplementary Materials) hosts the CCE-based snapshots of the final configuration for the corresponding systems.

While perfection in the form of an FCC crystal has been reported very recently from extremely long simulations on freely-jointed chains of hard spheres [52], it is the first time that an almost perfect HCP crystal, made of hard-sphere chains, in extended rod-like conformations, is observed. The easiness with which the crystal perfection is observed could be related to the nematic ordering exhibited by the rod-like chains. This trend, particularly compared to the fully flexible model, will be explored in more detail in future studies.

### 3.3. Local Order: Total Crystallinity

From the local order parameters $S^{X}$, the total degree of crystallinity, $\tau_{c}$, can be gauged for all the simulated semi-flexible systems. As in the previous analysis, the present results are compared against the ones of fully flexible (freely-jointed) analogs. Figure S5 (see Supplementary Materials) presents the evolution of the degree of crystallinity as a function of the MC steps at different packing densities, φ.

**Figure 10:**
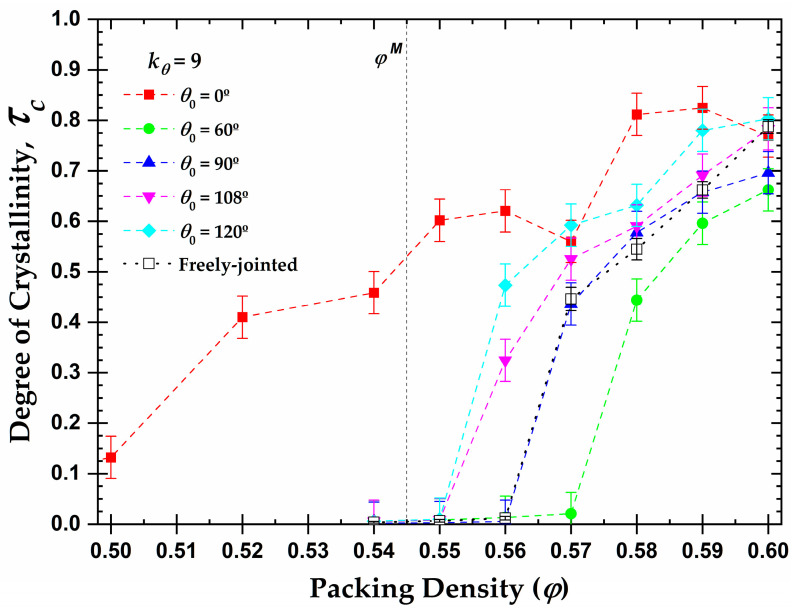
Degree of crystallinity, $\tau_{c}$, as a function of packing density, φ, and equilibrium bending angle, $\theta_{0}$, for 100-chains *N_av_* = 12 systems of semi-flexible chains with bending constant $k_{\theta }=9$ (filled symbols) and freely-jointed chains (open square). Dashed lines connecting points are used as visual support. Vertical dashed line corresponds to the melting transition ($\varphi_{monomers}^{M}\approx 0.545$ ) for monomeric hard spheres [29].

Based on these results and utilizing the value of the total crystallinity as established in the final, stable part of the MC simulation, we can further extract the one-dimensional phase diagram of local order, quantified by the degree of crystallinity, as a function of the packing density and equilibrium bending angle (Figure 10). It is worth keeping in mind here that the melting point for monomers is $\varphi_{monomers}^{M}=0.545,$ while freely-jointed chains of tangent hard spheres crystallize at higher volume fractions, $\varphi_{chains}^{M}$ (>0.56). Some important conclusions can be drawn from the diagram. First, all of the semi-flexible chain packings eventually crystallize, independently of the equilibrium angle. Second, the equilibrium angle profoundly affects the onset of the phase transition of the systems. For example, the rod-like chains present semi-crystalline and crystalline phases at volume fractions significantly lower than the melting point for freely-jointed chains, even lower than the one for monomeric hard spheres, due to the effect of the nematic (global) ordering that precedes the local order (as will be demonstrated in the continuation). On the other hand, chains with $\theta_{0}=60{}^{\circ}$ crystallize later than all of the other systems, as at φ=0.57 no crystal nucleation and growth is observed, even after $7\times 10^{11}$ MC steps. Finally, based on the above, the melting transition shows the trend: $\varphi_{chains}^{M}\left(0{}^{\circ}\right)<\varphi_{monomers}^{M}=0.545<\varphi_{chains}^{M}\left(120{}^{\circ}\right)<\varphi_{chains}^{M}\left(108{}^{\circ}\right)<\varphi_{chains}^{M}\left(90{}^{\circ}\right)=\varphi_{chains}^{M}\left(\mathrm{FJ}\right)<\varphi_{chains}^{M}\left(60{}^{\circ}\right)$.

In general, with the sole exception of the rod-like chains, the presence of constraints, related to bond geometry in the form of bonds or bending angles, increases the melting transition with respect to the monomeric analogs. Nevertheless, it is quite surprising that the systems with obtuse bending angles ($\theta_{0}=120\;\mathrm{and}\;108{}^{\circ}$) crystallize earlier, and the right angle ($\theta_{0}=90{}^{\circ}$) at a very similar volume fraction as the fully flexible chains, which are completely free of bending constraints. Obtuse angles are favored at high volume fractions as they minimize the local volume compared to acute ones. However, one should further consider the fact that only specific geometric arrangements of polymer chains are compatible with the sites of ideal FCC and HCP crystals. For example, the FCC and HCP share the bending angles of 0, 60, 90, and 120°, while the angles at 33.5 and 70.5° exist exclusively on the HCP crystal [52]. The lack of bending angles compatible with these crystals would require the adjacent sites to be covered by monomers belonging to other chains, imposing specific arrangements at the intermolecular level.

**Figure 11:**
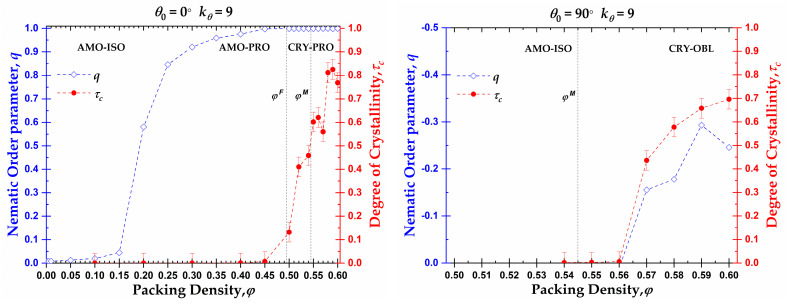
Orientational order parameter, *q* ((**left**) *y*-axis, blue color), and crystallinity, $\tau_{c}$ ((**right**) *y*-axis, red color), as a function of packing density, φ, for 100-chains *N_av_* = 12 systems of semi-flexible chains with bending constant $k_{\theta }=9$ and equilibrium bending angle $\theta_{0}=0{}^{\circ}$ (**left**) and $\theta_{0}=90{}^{\circ}$ (**right**). Dashed lines connecting points are used as visual support. Vertical dashed lines correspond to the freezing ($\varphi_{monomers}^{F}\approx 0.494$ ) and melting ($\varphi_{monomers}^{M}=0.545$ ) transition for monomeric hard spheres [29].

The phase behavior at the local (CCE-based crystallinity, $\tau_{c}$) and global (nematic orientational order parameter, *q*) levels as a function of the density for rod-like and right-angle chains is presented in Figure 11. As was explained previously, and based on the data of Figure 4, the rod-like chains present an isotropic-nematic transition at $\varphi^{nem}\approx 0.20$, a volume fraction significantly lower than the freezing point for monomeric hard spheres ($\varphi_{monomers}^{F}=0.494$), in accordance with the trends presented in [106,107]. At volume fractions close to the freezing point, the rod-like systems reach a practically perfect PRO phase. According to Shakirov and Paul [87], the rod-like chains self-arrange into a 2D hexagonal crystal structure in the plane perpendicular to the nematic director at a volume fraction φ=0.47, driven by 2D translational entropy. Thus, those 2D hexagonal crystal structures evolve into a 3D semi-crystalline phase of RHCP structures with the increase in concentration in the range between the freezing and melting point for monomeric hard spheres. After reaching the melting point for monomeric hard spheres, the nematic systems start to crystallize in the HCP, FCC, or mixed HCP/FCC structures, maintaining the nematic order. Thus, rod-like systems pack at high densities into Nematic Close Packed (NCP) structures, as was observed by independent researchers through MD simulations [85,86].

Based on the local and global phase behavior, the one-dimensional diagram as a function of the packing density can be split into three distinct regions, marked by XX-YY, where the first index XX corresponds to the local structure (XX = AMO or CRY) and the second index YY to the global structure (YY = ISO, PRO or OBL). Accordingly, for the rod-like chains studied here (*N_av_* = 12, $k_{\theta }=9$), we have the following approximate domains of phase behavior (left panel of Figure 11): (i) AMO-ISO (φ≤0.15), where the system is amorphous at the local level and isotropic at the global; (ii) AMO-PRO (0.20≤φ≤0.45), where the packing is amorphous locally and nematic globally; and (iii) CRY-PRO (0.50≤φ), where the system shows crystallinity in the form of HCP and FCC close packed morphologies of varied levels of perfection, and perfect nematic ordering at the global orientational level. Comparing the local and global trends, a delay is evident as the local order is established at volume fractions significantly higher than the ones for the ISO → PRO transition.

In the case of the right-angle chains ($\theta_{0}=90{}^{\circ}$), the phase behavior is quite different compared to the rod-like chains, as seen in the right panel of Figure 11. First, we should note that the small decrease in the long-range orientational order can be attributed to higher statistical uncertainty as the higher the packing density, and the closer to jamming, the more difficult the sampling, even when such advanced MC algorithms are employed. Accordingly, significantly longer simulations are required and the statistical uncertainty is higher. The right-angle systems do not crystallize until after they reach volume fractions higher than the melting point for monomeric hard spheres ($\varphi_{monomers}^{M}=0.545$). Then, the ISO → OBL transition occurs simultaneously to the crystal nucleation and growth. The formation of RHCP structures requires a semi-nematic OBL phase for the right-angle systems as the right-angle chain exists but is not dominant in the FCC and HCP crystals. Accordingly, for their creation, specific chain alignments are required. As a result, the local and global orders are simultaneously established, and no delay is observed. The phase diagram consists of only two regimes: (i) AMO-ISO (φ≤0.56), where the system is amorphous at the local level and isotropic at the global level; and (ii) CRY-OBL (φ>0.56), where the system shows RHCP morphologies and a nematic order of oblate mesogens.

## 4. Discussion and Conclusions

We present the results from extensive Monte Carlo simulations on the phase behavior of semi-flexible chains of tangent hard spheres as a function of the packing density and equilibrium bending angle while fixing the temperature, spring bending constant, and average chain length. The local structure is quantified through the Characteristic Crystallographic Element (CCE) norm, while the global structure is gauged through the nematic order parameter. A rich one-dimensional phase diagram as a function of packing density is identified where chains crystallize in close-packed morphologies, including random hexagonal close (RHCP) ones of single or multiple stacking directions, or in almost perfect HCP and FCC crystals in the case of rod-like chains. The analysis of the long-range orientational tensor reveals the formation of prolate mesogen, nematic mesophase (PRO) for rod-like chains at rather low volume fractions and of oblate mesogen, nematic mesophase (OBL) at high packing densities. Although all of the systems of semi-flexible chains crystallize, the equilibrium bending angle significantly affects the melting point. While equilibrium angles of 108° and 120° degrees favor crystallization compared to the freely-jointed model, chains with 90° show a behavior that almost coincides with the fully flexible chains, and the acute angle of 60° hinders crystallization, enforcing nucleation and growth to take place at higher concentrations.

In particular, for rod-like chains, three distinct regimes can be identified in the one-dimensional phase diagram, where a delay is observed between local and global self-organization: (i) AMO-ISO (φ≤0.15), where the system is amorphous at the local level and isotropic at the global; (ii) AMO-PRO 0.20 ≤ *φ* ≤ 0.45), where the packing is amorphous locally and nematic globally; and (iii) CRY-PRO (0.50≤φ), where the system shows crystallinity in the form of HCP and FCC close packed morphologies of varied levels of perfection and perfect nematic ordering at the global orientational level. Right-angle systems show a synchronous establishment of long-range nematic orientational order and formation of RHCP crystallites, thus splitting the behavior into two distinct regimes: (i) AMO-ISO (φ≤0.56), where the system is amorphous at the local level and isotropic at the global level; and (ii) CRY-OBL (φ>0.56), where the system is crystalline at the local level and shows a nematic mesophase of oblate mesogens at the long-range.

The present simulations are currently expanded to treat semi-flexible chains of tangent hard spheres in composites with nanofillers, under confinement, and in mixtures with different species in the form of linear chains and monomeric counterparts.

## Data Availability

The data presented in this study are openly available in Zenodo at https://zenodo.org/record/7554325#.Y8qE8u3ML8o, doi: 10.5281/zenodo.7554325.

## Supplementary Materials

The following are available online at https://www.mdpi.com/article/10.3390/polym15030551/s1, Figure S1: Snapshots of systems of semi-flexible chains of tangent hard spheres (*N_ch_* = 100, *N* = 12) at a packing density φ=0.10 with a bending constant $k_{\theta }=9$ and different equilibrium bending angles, $\theta_{0}$; Figure S2: Snapshots of the final configurations from MC simulations on semi-flexible chains of hard spheres at φ=0.60 and different equilibrium bending angles, $\theta_{0}$; Figure S3: Exponential moving average of the orientational order parameter, *q*, as a function of MC steps for 100-chains *N_av_* = 12 semi-flexible system with bending constant and equilibrium bending angle $\theta_{0}=0{}^{\circ}$ at different packing densities, φ; Figure S4: Final configuration for the 100-chains *N_av_* = 12 semi-flexible system chains with bending constant $k_{\theta }=9$ and equilibrium bending angle $\theta_{0}=0{}^{\circ}$ at φ=0.58 and 0.60; Figure S5: Degree of crystallinity, $\tau_{c}$, as a function of MC steps at different packing densities, φ.

## Abbreviations

The following abbreviations are used throughout the manuscript:

2D	Two dimensions
3D	Three dimensions
AMO	Amorphous
BCC	Body Centered Cubic
CB	Configurational Bias
CCAM	Chain-Connectivity-Altering Move
CCB	End-segment re-arrangement move
CCE	Characteristic Crystallographic Element (norm)
FCC	Face Centered Cubic
FIV	Fivefold
FJ	Freely-Jointed
HCP	Hexagonal Close Packed
HEX	Hexagonal
HON	Honeycomb
HS	Hard Sphere
ISO	Isotropic phase
MC	Monte Carlo
MD	Molecular Dynamics
NCP	Nematic Close Packed
NEM	Nematic mesophase
OBL	Oblate mesogen, Nematic mesophase
PBC	Periodic Boundary Condition
PEN	Pentagonal
PRO	Prolate mesogen, Nematic mesophase
RHCP	Random Hexagonal Close Packing
SQU	Square
TRI	Triangular

## References

[1] Flory P.J. (1989). Statistical Mechanics of Chain Molecules.

[2] deGennes P.G. (1980). Scaling Concepts in Polymer Physics.

[3] Mullin J.W. (2001). 8—Industrial Techniques and Equipment. Crystallization.

[4] Reiter G., Sommer J.U. (2008). Polymer Crystallization: Obervations, Concepts and Interpretations.

[5] Zhou J.H., Yang Y.S., Yang Y., Kim D.S., Yuan A., Tian X.Z., Ophus C., Sun F., Schmid A.K., Nathanson M. (2019). Observing Crystal Nucleation in Four Dimensions Using Atomic Electron Tomography. Nature.

[6] Hu Y.C., Tanaka H. (2020). Physical Origin of Glass Formation from Multicomponent Systems. Sci. Adv..

[7] Wang S.Z., Park S.S., Buru C.T., Lin H.X., Chen P.C., Roth E.W., Farha O.K., Mirkin C.A. (2020). Colloidal Crystal Engineering with Metal-Organic Framework Nanoparticles and DNA. Nat. Commun..

[8] Dijkstra M., Luijten E. (2021). From Predictive Modelling to Machine Learning and Reverse Engineering of Colloidal Self-Assembly. Nat. Mater..

[9] Zhang W., Mazzarello R., Wuttig M., Ma E. (2019). Designing Crystallization in Phase-Change Materials for Universal Memory and Neuro-Inspired Computing. Nat. Rev. Mater..

[10] Li M.M., Mangalore D.K., Zhao J.B., Carpenter J.H., Yan H.P., Ade H., Yan H., Mullen K., Blom P.W.M., Pisula W. (2018). Integrated Circuits Based on Conjugated Polymer Monolayer. Nat. Commun..

[11] Xia X.X., Lau T.K., Guo X.Y., Li Y.H., Qin M.C., Liu K., Chen Z., Zhan X.Z., Xiao Y.Q., Chan P.F. (2021). Uncovering the out-of-Plane Nanomorphology of Organic Photovoltaic Bulk Heterojunction by Gtsaxs. Nat. Commun..

[12] Alder B.J., Wainwright T.E. (1957). Phase Transition for a Hard Sphere System. J. Chem. Phys..

[13] Frenkel D. (1999). Entropy-Driven Phase Transitions. Phys. A.

[14] Pusey P.N., Vanmegen W. (1986). Phase-Behavior of Concentrated Suspensions of Nearly Hard Colloidal Spheres. Nature.

[15] Miskin M.Z., Jaeger H.M. (2013). Adapting Granular Materials through Artificial Evolution. Nat. Mater..

[16] Athanassiadis A.G., Miskin M.Z., Kaplan P., Rodenberg N., Lee S.H., Merritt J., Brown E., Amend J., Lipson H., Jaeger H.M. (2014). Particle Shape Effects on the Stress Response of Granular Packings. Soft Matter.

[17] Miskin M.Z., Jaeger H.M. (2014). Evolving Design Rules for the Inverse Granular Packing Problem. Soft Matter.

[18] Zou L.-N., Cheng X., Rivers M.L., Jaeger H.M., Nagel S.R. (2009). The Packing of Granular Polymer Chains. Science.

[19] Sacanna S., Irvine W.T.M., Chaikin P.M., Pine D.J. (2010). Lock and Key Colloids. Nature.

[20] Vutukuri H.R., Demirors A.F., Peng B., van Oostrum P.D.J., Imhof A., van Blaaderen A. (2012). Colloidal Analogues of Charged and Uncharged Polymer Chains with Tunable Stiffness. Angew. Chem. Int. Ed..

[21] Brown E., Nasto A., Athanassiadis A.G., Jaeger H.M. (2012). Strain Stiffening in Random Packings of Entangled Granular Chains. Phys. Rev. Lett..

[22] Feng L., Pontani L.L., Dreyfus R., Chaikin P., Brujic J. (2013). Specificity, Flexibility and Valence of DNA Bonds Guide Emulsion Architecture. Soft Matter.

[23] Allen M.P., Tildesley D.J. (2017). Computer Simulation of Liquids.

[24] Frenkel D., Smit B. (2002). Understanding Molecular Simulation: From Algorithms to Applications.

[25] Landau D.P., Binder K. (2014). A Guide to Monte Carlo Simulations in Statistical Physics.

[26] Rapaport D.C. (2004). The Art of Molecular Dynamics Simulation.

[27] Leach A. (2001). Molecular Modelling: Principles and Applications.

[28] Wood W.W., Jacobson J.D. (1957). Preliminary Results from a Recalculation of the Monte Carlo Equation of State of Hard Spheres. J. Chem. Phys..

[29] Hoover W.G., Ree F.H. (1968). Melting Transition and Communal Entropy for Hard Spheres. J. Chem. Phys..

[30] Rintoul M.D., Torquato S. (1996). Metastability and Crystallization in Hard-Sphere Systems. Phys. Rev. Lett..

[31] Rintoul M.D., Torquato S. (1996). Computer Simulations of Dense Hard-Sphere Systems. J. Chem. Phys..

[32] Bolhuis P.G., Frenkel D., Mau S.C., Huse D.A. (1997). Entropy Difference between Crystal Phases. Nature.

[33] Woodcock L.V. (1997). Entropy Difference between the Face-Centred Cubic and Hexagonal Close-Packed Crystal Structures. Nature.

[34] Bruce A.D., Wilding N.B., Ackland G.J. (1997). Free Energy of Crystalline Solids: A Lattice-Switch Monte Carlo Method. Phys. Rev. Lett..

[35] Pusey P.N., Vanmegen W., Bartlett P., Ackerson B.J., Rarity J.G., Underwood S.M. (1989). Structure of Crystals of Hard Colloidal Spheres. Phys. Rev. Lett..

[36] Gasser U., Weeks E.R., Schofield A., Pusey P.N., Weitz D.A. (2001). Real-Space Imaging of Nucleation and Growth in Colloidal Crystallization. Science.

[37] Zhu J.X., Li M., Rogers R., Meyer W., Ottewill R.H., Russell W.B., Chaikin P.M. (1997). Crystallization of Hard-Sphere Colloids in Microgravity. Nature.

[38] Verhaegh N.A.M., Vanduijneveldt J.S., Vanblaaderen A., Lekkerkerker H.N.W. (1995). Direct Observation of Stacking Disorder in a Colloidal Crystal. J. Chem. Phys..

[39] Petukhov A.V., Dolbnya I.P., Aarts D., Vroege G.J., Lekkerkerker H.N.W. (2003). Bragg Rods and Multiple X-Ray Scattering in Random-Stacking Colloidal Crystals. Phys. Rev. Lett..

[40] Vanmegen W., Underwood S.M. (1993). Change in Crystallization Mechanism at the Glass-Transition of Colloidal Spheres. Nature.

[41] Medvedev N.N., Bezrukov A., Shtoyan D. (2004). From Amorphous Solid to Defective Crystal. A Study of Structural Peculiarities in Close Packings of Hard Spheres. J. Struct. Chem..

[42] van Meel J.A., Frenkel D., Charbonneau P. (2009). Geometrical Frustration: A Study of Four-Dimensional Hard Spheres. Phys. Rev. E.

[43] O’Malley B., Snook I. (2003). Crystal Nucleation in the Hard Sphere System. Phys. Rev. Lett..

[44] Russo J., Tanaka H. (2016). Crystal Nucleation as the Ordering of Multiple Order Parameters. J. Chem. Phys..

[45] Lam M.A., Khusid B., Kondic L., Meyer W.V. (2021). Role of Diffusion in Crystallization of Hard-Sphere Colloids. Phys. Rev. E.

[46] Auer S., Frenkel D. (2001). Prediction of Absolute Crystal-Nucleation Rate in Hard-Sphere Colloids. Nature.

[47] Martelozzo V.C., Schofield A.B., Poon W.C.K., Pusey P.N. (2002). Structural Aging of Crystals of Hard-Sphere Colloids. Phys. Rev. E.

[48] Kegel W.K., Dhont J.K.G. (2000). "Aging" of the Structure of Crystals of Hard Colloidal Spheres. J. Chem. Phys..

[49] Dolbnya I.P., Petukhov A.V., Aarts D., Vroege G.J., Lekkerkerker H.N.W. (2005). Coexistence of Rhcp and Fcc Phases in Hard-Sphere Colloidal Crystals. Europhys. Lett..

[50] Pronk S., Frenkel D. (1999). Can Stacking Faults in Hard-Sphere Crystals Anneal out Spontaneously?. J. Chem. Phys..

[51] Luchnikov V., Gervois A., Richard P., Oger L., Troadec J.P. (2002). Crystallization of Dense Hard Sphere Packings—Competition of Hcp and Fcc Close Order. J. Mol. Liq..

[52] Herranz M., Foteinopoulou K., Karayiannis N.C., Laso M. (2022). Polymorphism and Perfection in Crystallization of Hard Sphere Polymers. Polymers.

[53] Espinosa J.R., Vega C., Valeriani C., Frenkel D., Sanz E. (2019). Heterogeneous Versus Homogeneous Crystal Nucleation of Hard Spheres. Soft Matter.

[54] Vega C., McBride C., MacDowell L.G. (2001). Liquid Crystal Phase Formation for the Linear Tangent Hard Sphere Model from Monte Carlo Simulations. J. Chem. Phys..

[55] Karayiannis N.C., Foteinopoulou K., Laso M. (2009). Entropy-Driven Crystallization in Dense Systems of Athermal Chain Molecules. Phys. Rev. Lett..

[56] Karayiannis N.C., Foteinopoulou K., Abrams C.F., Laso M. (2010). Modeling of Crystal Nucleation and Growth in Athermal Polymers: Self-Assembly of Layered Nano-Morphologies. Soft Matter.

[57] Karayiannis N.C., Foteinopoulou K., Laso M. (2013). Spontaneous Crystallization in Athermal Polymer Packings. Int. J. Mol. Sci..

[58] Karayiannis N.C., Foteinopoulou K., Laso M. (2015). The Role of Bond Tangency and Bond Gap in Hard Sphere Crystallization of Chains. Soft Matter.

[59] Ramos P.M., Herranz M., Foteinopoulou K., Karayiannis N.C., Laso M. (2021). Entropy-Driven Heterogeneous Crystallization of Hard-Sphere Chains under Unidimensional Confinement. Polymers.

[60] Ramos P.M., Herranz M., Martinez-Fernandez D., Foteinopoulou K., Laso M., Karayiannis N.C. (2022). Crystallization of Flexible Chains of Tangent Hard Spheres under Full Confinement. J. Phys. Chem. B.

[61] Sirota E.B. (2007). Polymer Crystallization: Metastable Mesophases and Morphology. Macromolecules.

[62] Sirota E.B., Singer D.M. (1994). Phase-Transitions among the Rotator Phases of the Normal-Alkanes. J. Chem. Phys..

[63] Sirota E.B., King H.E., Singer D.M., Shao H.H. (1993). Rotator Phases of the Normal Alkanes—An X-Ray-Scattering Study. J. Chem. Phys..

[64] Bourque A., Locker C.R., Rutledge G.C. (2016). Molecular Dynamics Simulation of Surface Nucleation During Growth of an Alkane Crystal. Macromolecules.

[65] Morthomas J., Fusco C., Zhai Z.Q., Lame O., Perez M. (2017). Crystallization of Finite-Extensible Nonlinear Elastic Lennard-Jones Coarse-Grained Polymers. Phys. Rev. E.

[66] Luo C.F., Kroger M., Sommer J.U. (2016). Entanglements and Crystallization of Concentrated Polymer Solutions: Molecular Dynamics Simulations. Macromolecules.

[67] Zhang W., Zou L. (2021). Molecular Dynamics Simulations of Crystal Nucleation near Interfaces in Incompatible Polymer Blends. Polymers.

[68] Isobe M., Krauth W. (2015). Hard-Sphere Melting and Crystallization with Event-Chain Monte Carlo. J. Chem. Phys..

[69] Zhang W., Larson R.G. (2018). Direct All-Atom Molecular Dynamics Simulations of the Effects of Short Chain Branching on Polyethylene Oligomer Crystal Nucleation. Macromolecules.

[70] Ramos J., Vega J.F., Sanmartin S., Martinez-Salazar J. (2016). Coarse-Grained Simulations on the Crystallization, Melting and Annealing Processes, of Short Chain Branched Polyolefins. Eur. Polym. J..

[71] Ramos J., Vega J.F., Martinez-Salazar J. (2018). Predicting Experimental Results for Polyethylene by Computer Simulation. Eur. Polym. J..

[72] Fall W.S., Baschnagel J., Lhost O., Meyer H. (2022). Role of Short Chain Branching in Crystalline Model Polyethylenes. Macromolecules.

[73] Sanmartin S., Ramos J., Vega J.F., Martinez-Salazar J. (2014). Strong Influence of Branching on the Early Stage of Nucleation and Crystal Formation of Fast Cooled Ultralong N-Alkanes as Revealed by Computer Simulation. Eur. Polym. J..

[74] Yi P., Locker C.R., Rutledge G.C. (2013). Molecular Dynamics Simulation of Homogeneous Crystal Nucleation in Polyethylene. Macromolecules.

[75] Ramos J., Martinez-Salazar J. (2011). Computer Modeling of the Crystallization Process of Single-Chain Ethylene/1-Hexene Copolymers from Dilute Solutions. J. Polym. Sci. Part B-Polym. Phys..

[76] Yamamoto T. (2022). Chiral Selecting Crystallization of Helical Polymers: A Molecular Dynamics Simulation for the Pom-Like Bare Helix. J. Chem. Phys..

[77] Yi P., Rutledge G.C. (2009). Molecular Simulation of Crystal Nucleation in N-Octane Melts. J. Chem. Phys..

[78] Romanos N.A., Theodorou D.N. (2010). Crystallization and Melting Simulations of Oligomeric Alpha 1 Isotactic Polypropylene. Macromolecules.

[79] Karayiannis N.C., Foteinopoulou K., Laso M. (2009). The Structure of Random Packings of Freely Jointed Chains of Tangent Hard Spheres. J. Chem. Phys..

[80] Karayiannis N.C., Malshe R., Kroger M., de Pablo J.J., Laso M. (2012). Evolution of Fivefold Local Symmetry During Crystal Nucleation and Growth in Dense Hard-Sphere Packings. Soft Matter.

[81] Karayiannis N.C., Malshe R., de Pablo J.J., Laso M. (2011). Fivefold Symmetry as an Inhibitor to Hard-Sphere Crystallization. Phys. Rev. E.

[82] Karayiannis N.C., Foteinopoulou K., Laso M. (2014). Twinning of Polymer Crystals Suppressed by Entropy. Symmetry.

[83] Ni R., Dijkstra M. (2013). Effect of Bond Length Fluctuations on Crystal Nucleation of Hard Bead Chains. Soft Matter.

[84] Luo C.F., Kroger M., Sommer J.U. (2017). Molecular Dynamics Simulations of Polymer Crystallization under Confinement: Entanglement Effect. Polymer.

[85] Nguyen H.T., Smith T.B., Hoy R.S., Karayiannis N.C. (2015). Effect of Chain Stiffness on the Competition between Crystallization and Glass-Formation in Model Unentangled Polymers. J. Chem. Phys..

[86] Nguyen H.T., Hoy R.S. (2016). Effect of Chain Stiffness and Temperature on the Dynamics and Microstructure of Crystallizable Bead-Spring Polymer Melts. Phys. Rev. E.

[87] Shakirov T., Paul W. (2018). Crystallization in Melts of Short, Semiflexible Hard Polymer Chains: An Interplay of Entropies and Dimensions. Phys. Rev. E.

[88] Shakirov T. (2019). Crystallisation in Melts of Short, Semi-Flexible Hard-Sphere Polymer Chains: The Role of the Non-Bonded Interaction Range. Entropy.

[89] Donald A.M., Windle A.H., Hanna S. (2006). Liquid Crystalline Polymers.

[90] Ciferri A. (1991). Liquid Crystallinity in Polymers.

[91] Meyer R.B., Ciferri A., Krigbaum W.R. (1982). Polymer Liquid Crystals.

[92] Kato T., Uchida J., Ichikawa T., Soberats B. (2018). Functional Liquid-Crystalline Polymers and Supramolecular Liquid Crystals. Polym. J..

[93] Li Y., Huang Q., Shi T., An L. (2006). Effects of Chain Flexibility on Polymer Conformation in Dilute Solution Studied by Lattice Monte Carlo Simulation. J. Phys. Chem. B.

[94] Lamura A., Burkhardt T.W., Gompper G. (2001). Semiflexible Polymer in a Uniform Force Field in Two Dimensions. Phys. Rev. E.

[95] Hsu H.-P., Paul W., Binder K. (2010). Polymer Chain Stiffness Vs. Excluded Volume: A Monte Carlo Study of the Crossover Towards the Worm-Like Chain Model. Europhys. Lett..

[96] Cifra P. (2004). Differences and Limits in Estimates of Persistence Length for Semi-Flexible Macromolecules. Polymer.

[97] Zierenberg J., Marenz M., Janke W. (2016). Dilute Semiflexible Polymers with Attraction: Collapse, Folding and Aggregation. Polymers.

[98] Auhl R., Everaers R., Grest G.S., Kremer K., Plimpton S.J. (2003). Equilibration of Long Chain Polymer Melts in Computer Simulations. J. Chem. Phys..

[99] Bulacu M., van der Giessen E. (2007). Molecular-Dynamics Simulation Study of the Glass Transition in Amorphous Polymers with Controlled Chain Stiffness. Phys. Rev. E.

[100] Plaza-Rivera C.O., Nguyen H.T., Hoy R.S. (2017). Isostaticity and the Solidification of Semiflexible Polymer Melts. Soft Matter.

[101] Onsager L. (1949). The Effects of Shape on the Interaction of Colloidal Particles. Ann. New York Acad. Sci..

[102] Bolhuis P., Frenkel D. (1997). Tracing the Phase Boundaries of Hard Spherocylinders. J. Chem. Phys..

[103] Khokhlov A.R., Semenov A.N. (1985). On the Theory of Liquid-Crystalline Ordering of Polymer-Chains with Limited Flexibility. J. Stat. Phys..

[104] Khokhlov A.R., Semenov A.N. (1982). Liquid-Crystalline Ordering in the Solution of Partially Flexible Macromolecules. Phys. A.

[105] Khokhlov A.R., Semenov A.N. (1981). Liquid-Crystalline Ordering in the Solution of Long Persistent Chains. Phys. A.

[106] Yethiraj A., Fynewever H. (1998). Isotropic to Nematic Transition in Semiflexible Polymer Melts. Mol. Phys..

[107] Fynewever H., Yethiraj A. (1998). Phase Behavior of Semiflexible Tangent Hard Sphere Chains. J. Chem. Phys..

[108] Jaffer K.M., Opps S.B., Sullivan D.E., Nickel B.G., Mederos L. (2001). The Nematic-Isotropic Phase Transition in Semiflexible Fused Hard-Sphere Chain Fluids. J. Chem. Phys..

[109] Ivanov V.A., Martemyanova J.A., Rodionova A.S., Stukan M.R. (2013). Computer Simulation of Stiff-Chain Polymers. Polym. Sci. Ser. C.

[110] Tang X.L., Chen W., Li L.B. (2019). The Tough Journey of Polymer Crystallization: Battling with Chain Flexibility and Connectivity. Macromolecules.

[111] Semenov A.N., Subbotin A.V. (1989). Phase Equilibria in Mixtures of Rigid Chain Polymers. Polym. Sci. U.S.S.R..

[112] Egorov S.A., Milchev A., Virnau P., Binder K. (2016). Semiflexible Polymers under Good Solvent Conditions Interacting with Repulsive Walls. J. Chem. Phys..

[113] Egorov S.A., Milchev A., Binder K. (2016). Semiflexible Polymers in the Bulk and Confined by Planar Walls. Polymers.

[114] Kos P.I., Ivanov V.A., Chertovich A.V. (2021). Crystallization of Semiflexible Polymers in Melts and Solutions. Soft Matter.

[115] Roy S., Chen Y.L. (2021). Rich Phase Transitions in Strongly Confined Polymer-Nanoparticle Mixtures: Nematic Ordering, Crystallization, and Liquid-Liquid Phase Separation. J. Chem. Phys..

[116] Milchev A., Egorov S.A., Binder K., Nikoubashman A. (2018). Nematic Order in Solutions of Semiflexible Polymers: Hairpins, Elastic Constants, and the Nematic-Smectic Transition. J. Chem. Phys..

[117] Milchev A., Egorov S.A., Midya J., Binder K., Nikoubashman A. (2021). Blends of Semiflexible Polymers: Interplay of Nematic Order and Phase Separation. Polymers.

[118] Purdy K.R., Varga S., Galindo A., Jackson G., Fraden S. (2005). Nematic Phase Transitions in Mixtures of Thin and Thick Colloidal Rods. Phys. Rev. Lett..

[119] Russo P.S., Cao T. (1988). Phase-Behavior in a Ternary Rod Coil Solvent System—Poly(Gamma-Benzyl-Alpha,L-Glutamate) Nylon-6/M-Cresol. Mol. Cryst. Liq. Cryst..

[120] Dutta D., Fruitwala H., Kohli A., Weiss R.A. (1990). Polymer Blends Containing Liquid-Crystals—A Review. Polym. Eng. Sci..

[121] Egorov S.A., Milchev A., Nikoubashman A., Binder K. (2021). Phase Separation and Nematic Order in Lyotropic Solutions: Two Types of Polymers with Different Stiffnesses in a Common Solvent. J. Phys. Chem. B.

[122] Midya J., Egorov S.A., Binder K., Nikoubashman A. (2019). Phase Behavior of Flexible and Semiflexible Polymers in Solvents of Varying Quality. J. Chem. Phys..

[123] Dennison M., Dijkstra M., van Roij R. (2011). The Effects of Shape and Flexibility on Bio-Engineered Fd-Virus Suspensions. J. Chem. Phys..

[124] Dennison M., Dijkstra M., van Roij R. (2011). Phase Diagram and Effective Shape of Semiflexible Colloidal Rods and Biopolymers. Phys. Rev. Lett..

[125] Dietz J.D., Hoy R.S. (2020). Two-Stage Athermal Solidification of Semiflexible Polymers and Fibers. Soft Matter.

[126] Hoy R.S. (2017). Jamming of Semiflexible Polymers. Phys. Rev. Lett..

[127] Herranz M., Martínez-Fernández D., Ramos P.M., Foteinopoulou K., Karayiannis N.C., Laso M. (2021). Simu-D: A Simulator-Descriptor Suite for Polymer-Based Systems under Extreme Conditions. Int. J. Mol. Sci..

[128] Karayiannis N.C., Laso M. (2008). Monte Carlo Scheme for Generation and Relaxation of Dense and Nearly Jammed Random Structures of Freely Jointed Hard-Sphere Chains. Macromolecules.

[129] Ramos P.M., Karayiannis N.C., Laso M. (2018). Off-Lattice Simulation Algorithms for Athermal Chain Molecules under Extreme Confinement. J. Comput. Phys..

[130] Karayiannis N.C., Foteinopoulou K., Laso M. (2013). Jamming and Crystallization in Athermal Polymer Packings. Philos. Mag..

[131] Foteinopoulou K., Karayiannis N.C., Laso M. (2015). Monte Carlo Simulations of Densely-Packed Athermal Polymers in the Bulk and under Confinement. Chem. Eng. Sci..

[132] Karayiannis N.C., Foteinopoulou K., Laso M. (2009). The Characteristic Crystallographic Element Norm: A Descriptor of Local Structure in Atomistic and Particulate Systems. J. Chem. Phys..

[133] Ramos P.M., Herranz M., Foteinopoulou K., Karayiannis N.C., Laso M. (2020). Identification of Local Structure in 2-D and 3-D Atomic Systems through Crystallographic Analysis. Crystals.

[134] Siepmann J.I., Frenkel D. (1992). Configurational Bias Monte-Carlo—A New Sampling Scheme for Flexible Chains. Mol. Phys..

[135] Laso M., de Pablo J.J., Suter U.W. (1992). Simulation of Phase-Equilibria for Chain Molecules. J. Chem. Phys..

[136] Andrienko D. (2018). Introduction to Liquid Crystals. J. Mol. Liq..

[137] Nye J.F. (2010). Physical Properties of Crystals: Their Representation by Tensors and Matrices.

[138] Malgrange C., Ricolleau C., Schlenker M. (2014). Symmetry and Physical Properties of Crystals.

[139] Giacovazzo C., Monaco H.L., Artioli G., Viterbo D., Ferraris G., Gilli G., Zanotti G., Gatti M. (2005). Fundamentals of Crystallography.

[140] Laso M., Jimeno N. (2020). Representation Surfaces for Physical Properties of Materials: A Visual Approach to Understanding Anisotropic Materials.

[141] Humphrey W., Dalke A., Schulten K. (1996). Vmd: Visual Molecular Dynamics. J. Mol. Graph. Modell..

[142] Hoy R.S., Karayiannis N.C. (2013). Simple Model for Chain Packing and Crystallization of Soft Colloidal Polymers. Phys. Rev. E.

