# Supplementary material for: Local and Global Order in Dense Packings of Semi-Flexible Polymers of Hard Spheres
Source: Polymers (Basel). 2023 Jan 20;15(3):551. doi: 10.3390/polym15030551 (PMC9919756; doi:10.3390/polym15030551)
Supplement: Supplementary file 1 [file polymers-15-00551-s001.zip › polymers-2160116-supplementary.pdf]

polymers

MDPI

Supplementary Materials: Local and Global Order in Dense Packings of Semiflexible Polymers of Hard Spheres

Daniel Martínez-Fernández, Miguel Herranz, Katerina Foteinopoulou, Nikos Ch. Karayiannis * and Manuel Laso

Institute for Optoelectronic Systems and Microtechnology (ISOM) and Escuela Técnica Superior de Ingenieros Industriales (ETSII), Universidad Politécnica de Madrid (UPM), José Gutierrez Abascal 2, 28006 Madrid, Spain
* Correspondence: n.karayiannis@upm.es ; Tel.: +34-910677318 (N.C.K.)

Figure S1 hosts panels of computer-generated configurations of the semi-flexible systems at dilute conditions ($\varphi = 0.10$).

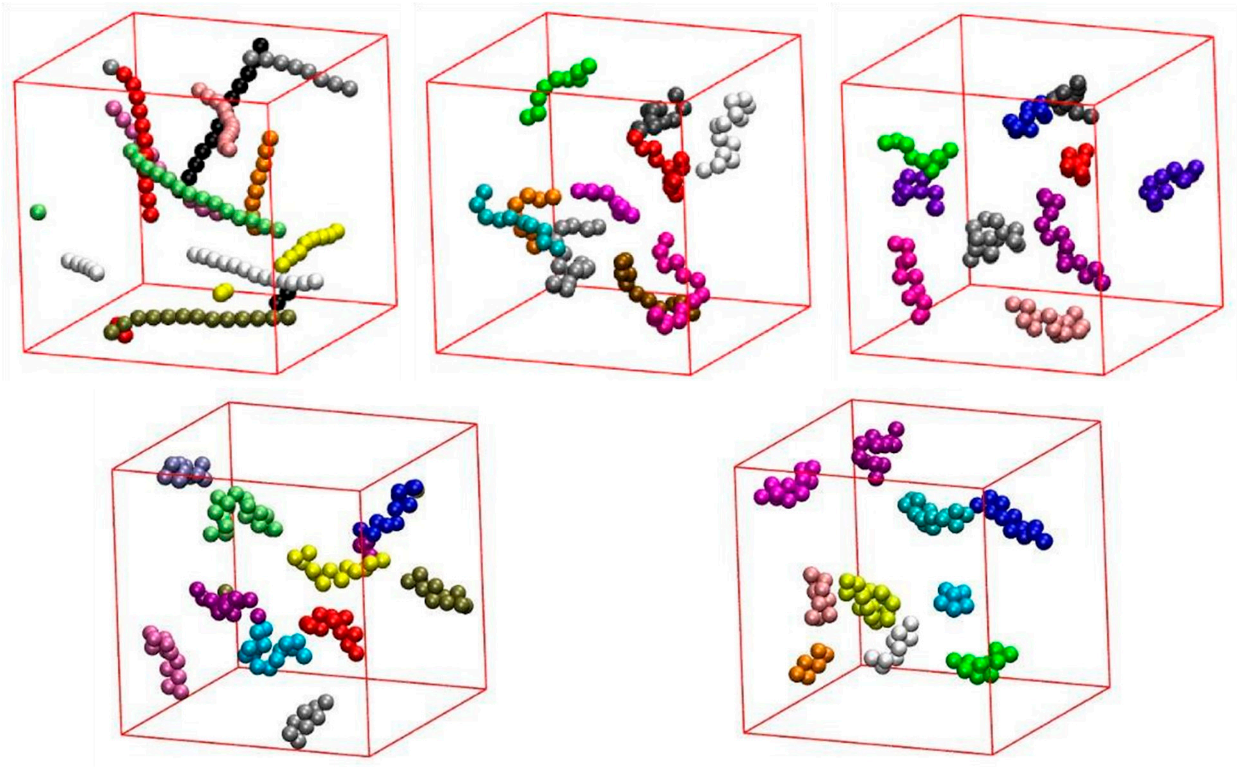

Figure S1. Snapshots of systems of semi-flexible chains of tangent hard spheres ($N_{ch} = 100$, $N = 12$) at a packing density $\varphi = 0.10$ with a bending constant $k_\theta = 9$ and different equilibrium bending angles, θ_0: (top, left) 0°, (top, middle) 60°, (top, right) 90°, (bottom, left) 108°, and (bottom, right) 120°. Only 10 chains are shown for clarity. Monomers are colored according to their parent chain. Sphere monomers are shown with the coordinates of their centers subjected to periodic boundary conditions. Image created with VMD visualization software [141]. Individual panels are also available as stand-alone, interactive, 3D images (in Supporting Information).

Figure S2 shows computer-generated snapshots of bulk system configurations at the end of the simulation for the studied semi-flexible hard-sphere systems at a packing density $\varphi = 0.60$. Through this preliminary visualization with monomers color-coded according to the parent chain, self-organization at the level of the local structure is clearly suggested in some of these systems when semi-flexible systems reach high packing densities, similar to that observed for freely-jointed (fully flexible) chains [55–57,79]. The degree of organization or crystallization and the type of structures of these systems are more thoroughly studied in posterior subsections through the CCE norm descriptor.

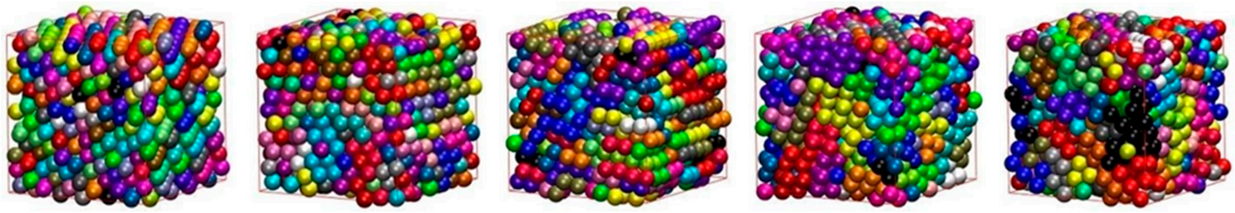

Figure S2. Snapshots of the final configurations from MC simulations on semi-flexible chains of hard spheres at φ = 0.60 and different equilibrium bending angles (from left to right), θ_0= 0, 60, 90, 108, and 120°. Monomers are colored according to the parent chain. Coordinates of monomers are subjected to periodic boundary conditions in all dimensions. Images created with VMD visualization software [141]. Individual panels are also available as stand-alone, interactive, 3D images (in Supporting Information).

The evolution of the simple running average of the orientational order parameter, q, for rod-like chains ($\theta_0 = 0°$) is presented in Figure S3 as a function of MC steps at various packing densities. Due to the unentangled, initial configurations of the systems, a very fast transition to the final degrees of orientational ordering is observed. An increase in packing density, φ, is associated with an increase in the degree of alignment, q, of the rod-like chains, producing an isotropic-nematic transition in the interval $0.15 \leq \varphi \leq 0.20$. Along with the increase of the orientational order parameter, the rod-like systems become more stable in their respective phases.

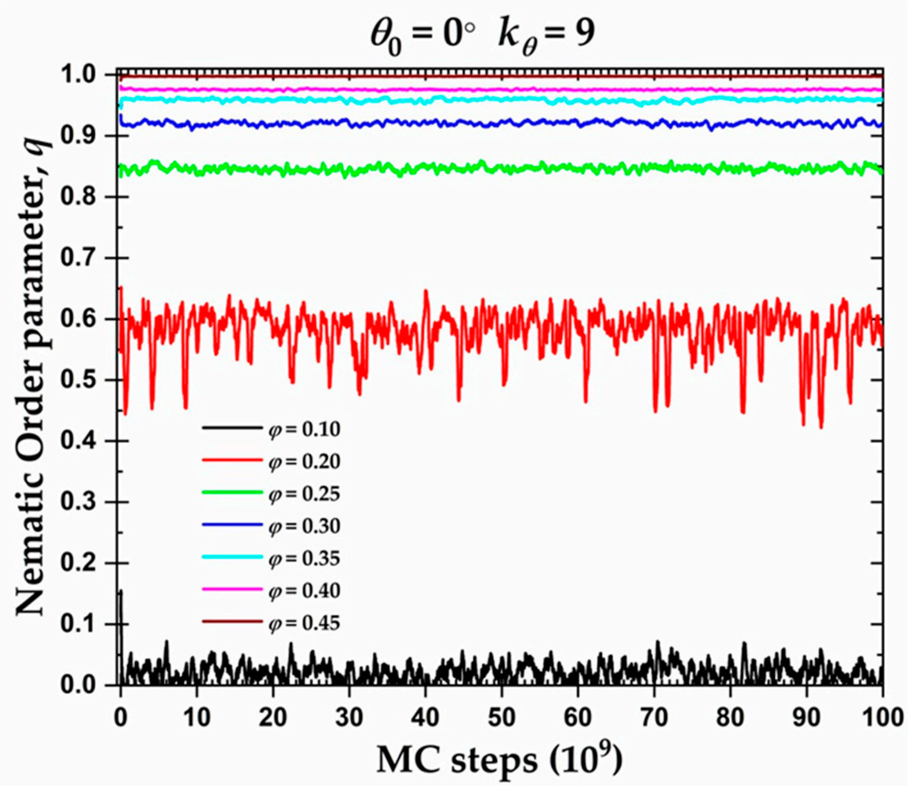

Figure S3. Exponential moving average of the orientational order parameter, q, as a function of MC steps for 100-chains N_{av} = 12 semi-flexible system with bending constant k_θ = 9 and equilibrium bending angle $\theta_0 = 0°$ at different packing densities, φ.

Figure S4 hosts the CCE-based snapshots for the final configuration of the rod-like chains (θ_0= 0°) at φ = 0.58 and 0.60. During the crystal nucleation and growth, the re-arrangement of the monomers gives rise to the FCC and HCP crystals, respectively, with a high degree of crystallinity.

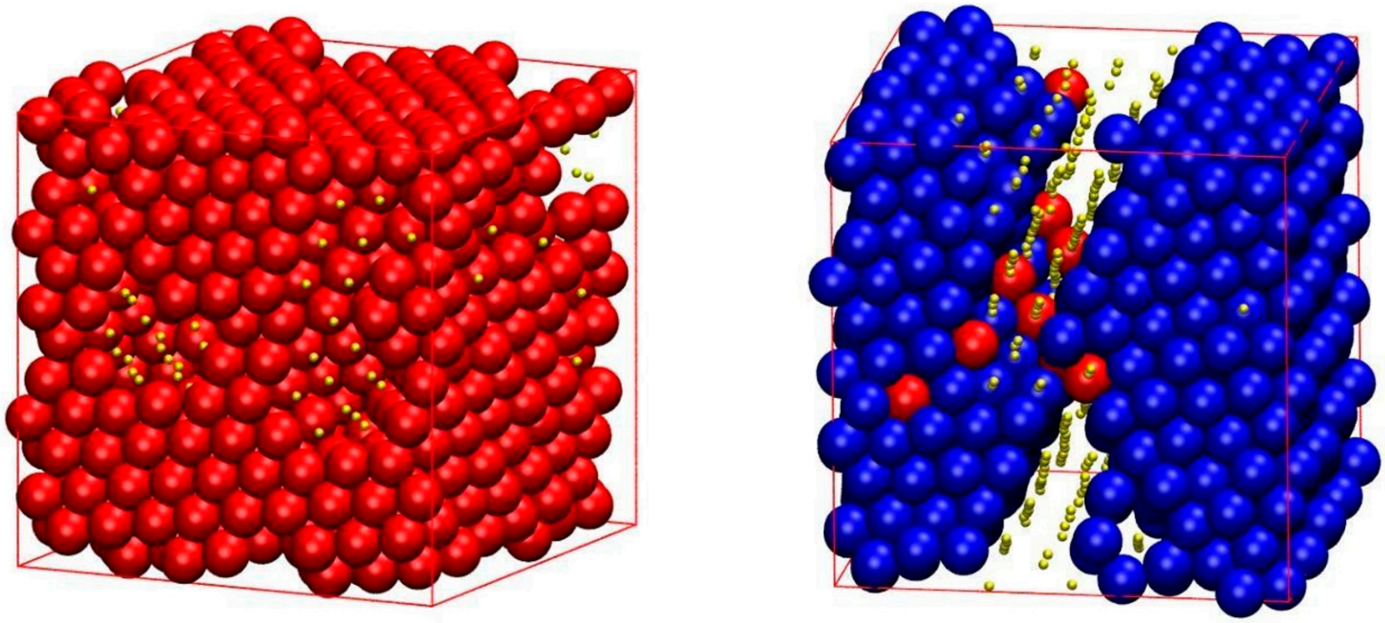

Figure S4. Final configuration for the 100-chains N_{av} = 12 semi-flexible system chains with bending constant k_θ = 9 and equilibrium bending angle θ_0 = 0° at φ = 0.58 (left panel) and 0.60 (right panel). Monomers are color-coded according to CCE norm. Blue, red, and green correspond to HCP-, FCC-, and FIV-like sites, respectively. Amorphous (AMO) sites are colored in yellow and are shown with reduced dimensions for clarity. Images created with VMD visualization software [141]. Individual panels are also available as standalone, interactive, 3D images (in Supplementary Materials).

Figure S5 presents the evolution of the degree of crystallinity as a function of MC steps at different packing densities, φ. All reported packing densities represented in the vicinity of the melting point for chains of tangent hard spheres, $\varphi_c{}^{M}{}_{hains}$ (> 0.56) [55,56,58]. The rod-like chains (θ_0 = 0°) present semi-crystalline and crystalline phases at volume fractions significantly lower than the melting point of freely-jointed chains but also of monomeric hard spheres, due to the effect of the nematic ordering. For the semi-flexible chains with θ_0 = 60°, crystallization at φ = 0.57 is not observed in the corresponding graph. This behavior could be because chain conformations produced present high constraints against the crystallization at this density or the system requires more simulation time, due to those constraints, to reach the phase transition.

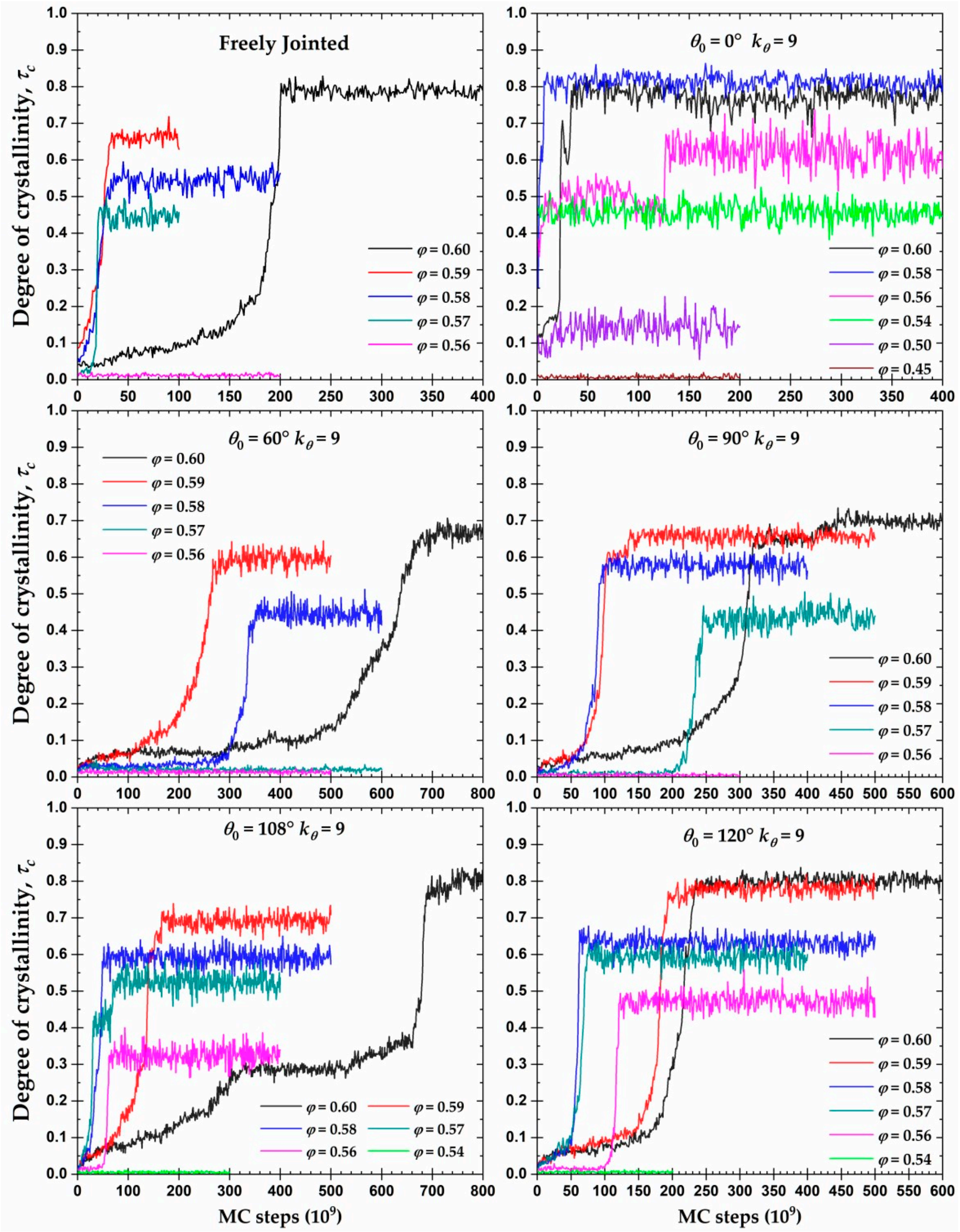

Figure S5. Degree of crystallinity, τ_c, as a function of MC steps at different packing densities, φ, for 100 chains, $N_{av} = 12$ systems of freely-jointed chains and semi-flexible chains with bending constant $k_\theta = 9$ and equilibrium bending angle θ_0= 0, 60, 90, 108, and 120°.
